# Tuning Nanographene-Enhanced Raman Scattering for Rapid Label-Free Detection of Amino Acids

**DOI:** 10.1021/acsami.4c08298

**Published:** 2024-09-24

**Authors:** Neha Sharma, Muhammad Hussnain Akmal, Ryoto Yura, Seyyed Mojtaba Mousavi, Darwin Kurniawan, Yoshiyuki Nonoguchi, Wei-Hung Chiang

**Affiliations:** † Department of Chemical Engineering, National Taiwan University of Science and Technology, Taipei 10607, Taiwan; ‡ Faculty of Materials Science and Engineering, Kyoto Institute of Technology, Kyoto 606-8585, Japan; § Sustainable Electrochemical Energy Development (SEED) Center, National Taiwan University of Science and Technology, Taipei City 10607, Taiwan

**Keywords:** plasmas, band gap tuning, graphene quantum dots, Raman scattering, amino acids, biosensors

## Abstract

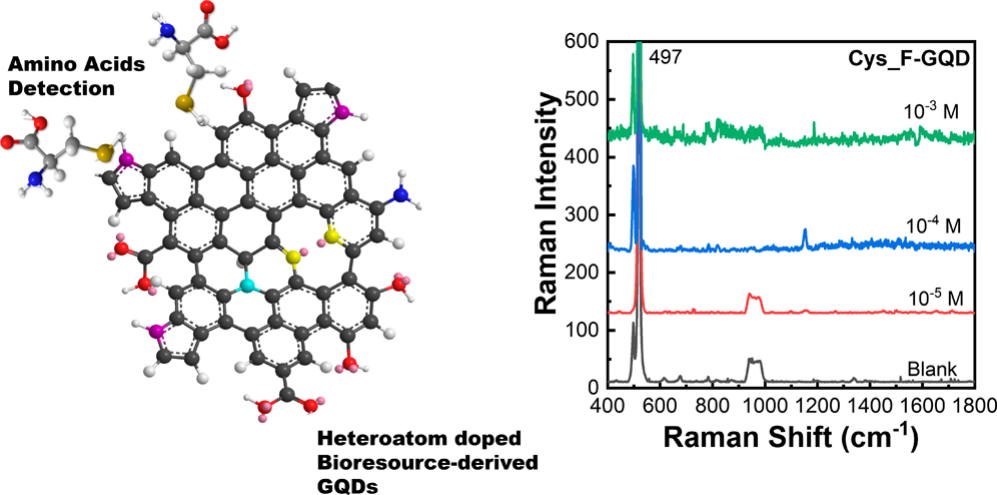

The rapid and sensitive detection of amino acids is important not only for fundamental studies but also for the establishment of a healthy society. However, conventional detection methods have been hampered by the difficulties of low sensitivity, long sampling and detection times, and expensive operation and instruments. Here, we report the plasma engineering of bioresource-derived graphene quantum dots (GQDs) as surface-enhanced Raman scattering (SERS)-active materials for the rapid and sensitive detection of amino acids. Surface-functionalized GQDs with tuned structures and band gaps were synthesized from earth-abundant bioresources by using reactive microplasmas under ambient conditions. Detailed microscopy and spectroscopy studies indicate that the SERS properties of the synthesized GQDs can be tuned by controlling the band gaps of synthesized GQDs. The plasma-synthesized metal-free GQDs with surface functionalities showed improved SERS properties for rapid amino acid detection with low detection limits of 10^–5^ M for tyrosine and phenylalanine. Theoretical calculations suggest that charge transfer between GQDs and amino acids can enhance the SERS response of the GQDs. Our work provides insights into the controlled engineering of SERS-active nanographene-based materials using the plasma-enhanced method.

## Introduction

Amino acids serve as vital building blocks for proteins, enzymes, and antibodies, which are crucial for the intricate working of biological systems. Their precise detection holds immense significance in biomedical diagnostics, environmental monitoring, and pharmaceuticals, as they are essential for synthesizing key metabolites vital for human health.^1−5^ While current technologies for amino acid detection, such as mass spectroscopy-enhanced,^6^ liquid chromatography,^7^ and electrochemical methods,^3,8−10^ have been developed, they often required specialized, time-consuming procedures, high costs, and complex sample preparation.^2−4,11^ These limitations hinder their widespread application in routine diagnostics and monitoring. Surface-enhanced Raman scattering (SERS) is a promising method for molecular detection with exceptional sensitivity and broad applications in chemical identification, biomolecule sensing, clinical analysis, food safety, disease and virus detection, and bioimaging.^12,13^ Traditionally, SERS has been achieved using plasmonic metals such as gold (Au) and silver (Ag) nanoparticles (NPs) due to their strong electromagnetic (EM)-induced Raman enhancement.^14−18^ However, the high cost of Au and Ag and poor stability in biological samples make it challenging to develop low-cost, highly sensitive, and stable SERS-based biomedical diagnostics and imaging. Consequently, there is a growing need for precious metal-free SERS-active materials for advancing SERS-based technologies. The new metal-free SERS-active material must offer excellent properties, including superior biocompatibility, reduced toxicity, and uniform signal reproducibility. Such features are critical for enabling label-free therapeutic drug monitoring and biomolecule detection, leading to more accessible and efficient diagnostic tools.

Graphene quantum dots (GQDs) represent a new class of nanographene materials renowned for their versatile applications in sensing, bioimaging, drug delivery, tissue engineering, catalysis, and energy storage.^19^ The unique electronic structure of GQDs, including a high density of electronic states and quantum confinement effects, enhances Raman signals through strong resonance effects and charge transfer interactions with analyte molecules. Their large surface area provides numerous active sites for adsorption, increasing the intensity of the SERS signal. Additionally, defects and functional groups in GQDs interact with biomolecules, enhancing selectivity and sensitivity, while their biocompatibility and chemical stability make them ideal for biological sensing application.^20−23^ These attributes coupled with property combination such as photostability and tunable charge mobility render GQDs to be promising as effective SERS-active materials for precise and sensitive molecular detection. The versatility of GQDs extends across a wide spectrum of applications, although their role as semiconductor substrates for SERS has conventionally relied on a charge transfer mechanism for signal enhancement. However, recent research has challenged this paradigm, suggesting that GQDs may exhibit surface plasmon resonance properties akin to those of metal nanoparticles (NPs).^24−27^ Yet, further detailed investigations are necessary to fully understand and quantify the potential contribution of the electromagnetic mechanism of GQDs. This intriguing revelation indicates the potential integration of the electromagnetic mechanism into the SERS enhancement repertoire of GQDs, thereby elevating their standing in the realm of advanced materials for cutting-edge applications. However, the precise control of the size and band gap of GQDs is crucial for optimizing their properties and applications. Achieving this level of control using conventional methods, such as chemical deposition, pulsed laser ablation, and hydrothermal methods, can be challenging due to the complex processes involved in the synthesis of GQDs.^28^ The current methods for synthesizing GQDs have limitations in controlling band gap tuning parameters effectively. Chemical synthesis routes may lead to variations in the size and band gap, posing challenges in achieving precise and homogeneous GQD structures.^29−31^

**Scheme 1:**
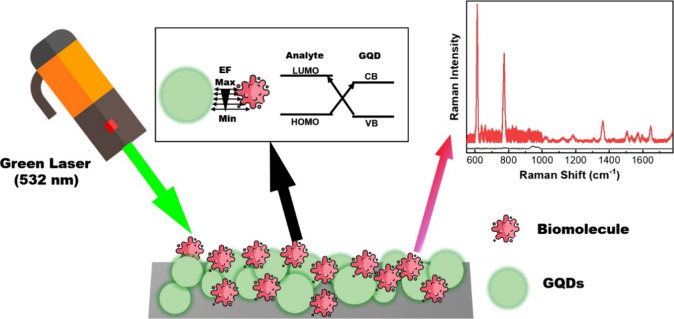
Plasma Engineering of Bioresource-Derived GQDs as the Precious Metal-Free SERS-Active Materials for Rapid and Sensitive Detection of Amino Acids Surface-functionalized GQDs with controlled structures and band gaps were initially synthesized from earth-abundant bioresources using reactive microplasmas under ambient conditions. The probe amino acids were attracted onto the surfaces of synthesized GQDs by the π–π* interaction. Charge transfer (CT) between GQDs and amino acids is found to be the factor enhancing the Raman scattering. The plasma-synthesized metal-free GQDs show enhanced Raman scattering. The plasma-synthesized metal-free GQDs show enhanced SERS properties for rapid and sensitive amino acid detection.

Herein, we report the plasma engineering of bioresource-derived GQDs as SERS-active materials for the rapid and sensitive detection of amino acids (Scheme 1). Nonthermal plasma-based technologies, especially microplasmas, have been explored as promising and effective methods for synthesizing metal nanoparticles, semiconductor quantum dots, and graphene-based nanomaterials.^19,32−35^ It is also possible to produce GQDs from bioresource precursors via microplasma synthesis.^36,37^ In this work, GQDs were synthesized from earth-abundant bioresource materials, including lignin, starch, chitosan, citric acid, and fructose, as carbon precursors using a microplasma-induced electrochemical method under ambient conditions. Detailed spectroscopy and microscopy characterization indicated that the band gaps of the plasma-synthesized GQDs could be controlled by adjusting the precursor chemical structures and plasma conditions. The plasma-synthesized GQDs with surface functionalities showed improved SERS properties for rapid amino acid detection, with low detection limits of 10^–5^ M for tyrosine and phenylalanine. Theoretical calculations suggest that charge transfer (CT) between the GQD and amino acids can enhance Raman scattering. Our work not only provides a new technology to prepare band gap-controlled nanomaterials but also an understanding of the SERS properties of nonprecious metal-based nanostructures.

## Results and Discussion

### Synthesis and Characterization of GQDs

**Figure 1:**
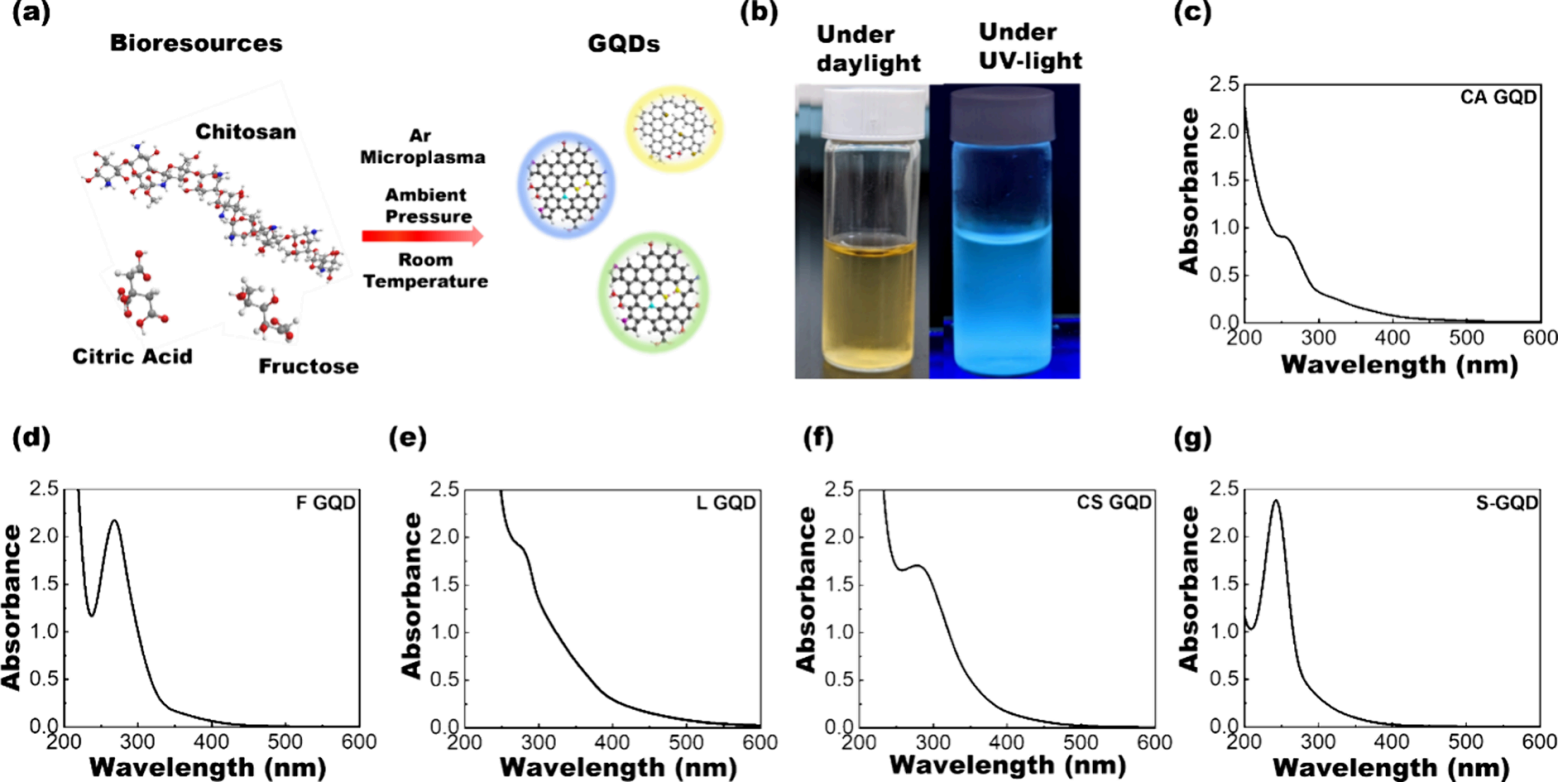
Plasma synthesis and absorbance study of bioresource-derived GQDs. (a) Schematic illustration of GQD synthesis from bioresources. (b) Visual representation of the GQD solution after microplasma treatment under both daylight and UV-light conditions. Absorption spectra of (c) CA-derived GQDs, (d) F-derived GQDs, (e) L-derived GQDs, (f) CS-derived GQDs, and (g) S-derived GQDs.

GQDs were synthesized from different bioresources, including lignin, chitosan, citric acid, fructose, and starch, using a customized microplasma reactor under ambient conditions (Figure 1a). The GQDs synthesized from lignin, chitosan, citric acid, fructose, and starch are denoted as L-GQD, CS-GQD, CA-GQD, F-GQD, and S-GQD, respectively. Details of the reactor and synthesis procedure can be found in our previous work^38^ and in the Supporting Information, respectively. After plasma synthesis, the GQDs exhibited a notable fluorescence emission under UV-light irradiation (Figure 1b). UV–vis absorbance spectroscopy was used to probe the optical properties of the synthesized samples. The UV–vis absorbance spectra (Figure 1c–g) show distinct absorption bands between 250 and 300 nm, which are attributed to the π–π* transition of sp^2^-hybridized graphene domains.^39,40^ The intensified absorption peaks of the CS-GQD and L-GQD can be attributed to doping with electron-donating nitrogen (N) and sulfur (S) atoms.^41,42^

**Figure 2:**
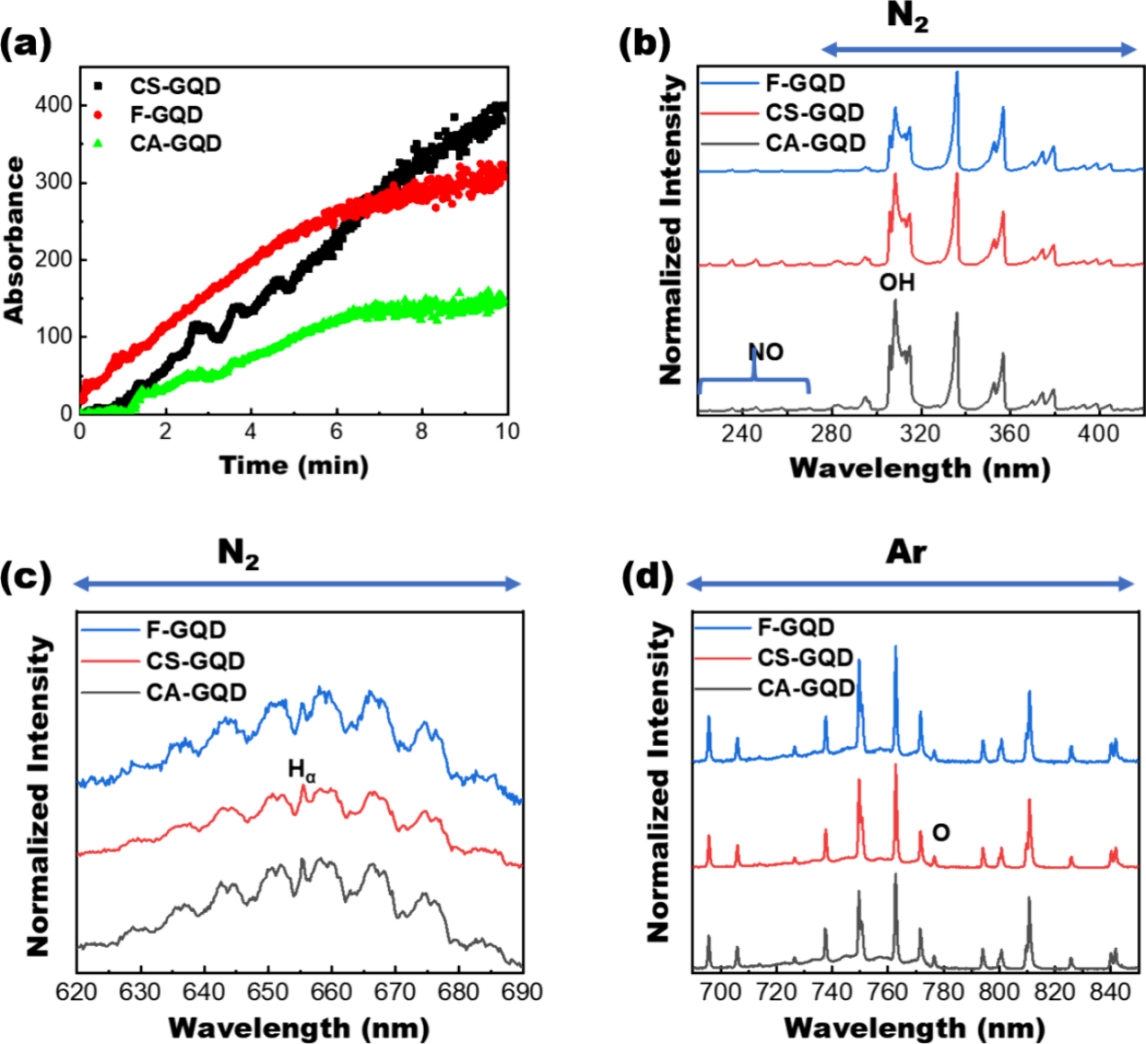
*In situ* absorption spectroscopy and OES study of GQD synthesis. (a) *In situ* absorbance for CS-GQD, F-GQD, and CA-GQD with different times. (b–d) Different sections of the OES spectra of GQD synthesis from different precursors using microplasmas.

*In situ* absorption spectroscopy and optical emission spectroscopy (OES) were used to study the growth of the GQDs under plasma (Figure 2). Figure 2 presents the *in situ* UV absorption spectra and *in situ* OES for three GQDs (one doped GQD and two undoped GQDs) as a representative to understand the growth of both doped and undoped GQDs during microplasma synthesis. The UV absorbance continuously increased during plasma synthesis, suggesting that the formation of GQDs was promoted under plasma conditions (Figure 2a). The OES spectra collected from the gas-phase plasmas indicate several excited species, including molecular NO bands below 272 nm, OH bands spanning 306–309 nm, the N2 second positive system extending from 280 to 450 nm, a faint Hα line proximal to the N2 first positive system at approximately 655.3 nm, atomic O transitions (2s^2^2p^3^3s-2s2sp^3^3p) manifesting around 776.3 nm, and Ar lines distributed between 690 and 850 nm (Figure 2b–d). These reactive radicals and solvated electrons can dissociate bioresource precursors to generate hydrocarbon moieties via cleavage of the precursors.^43−45^ Subsequently, GQDs can be nucleated and grown via a plasma synthesis.

**Figure 3:**
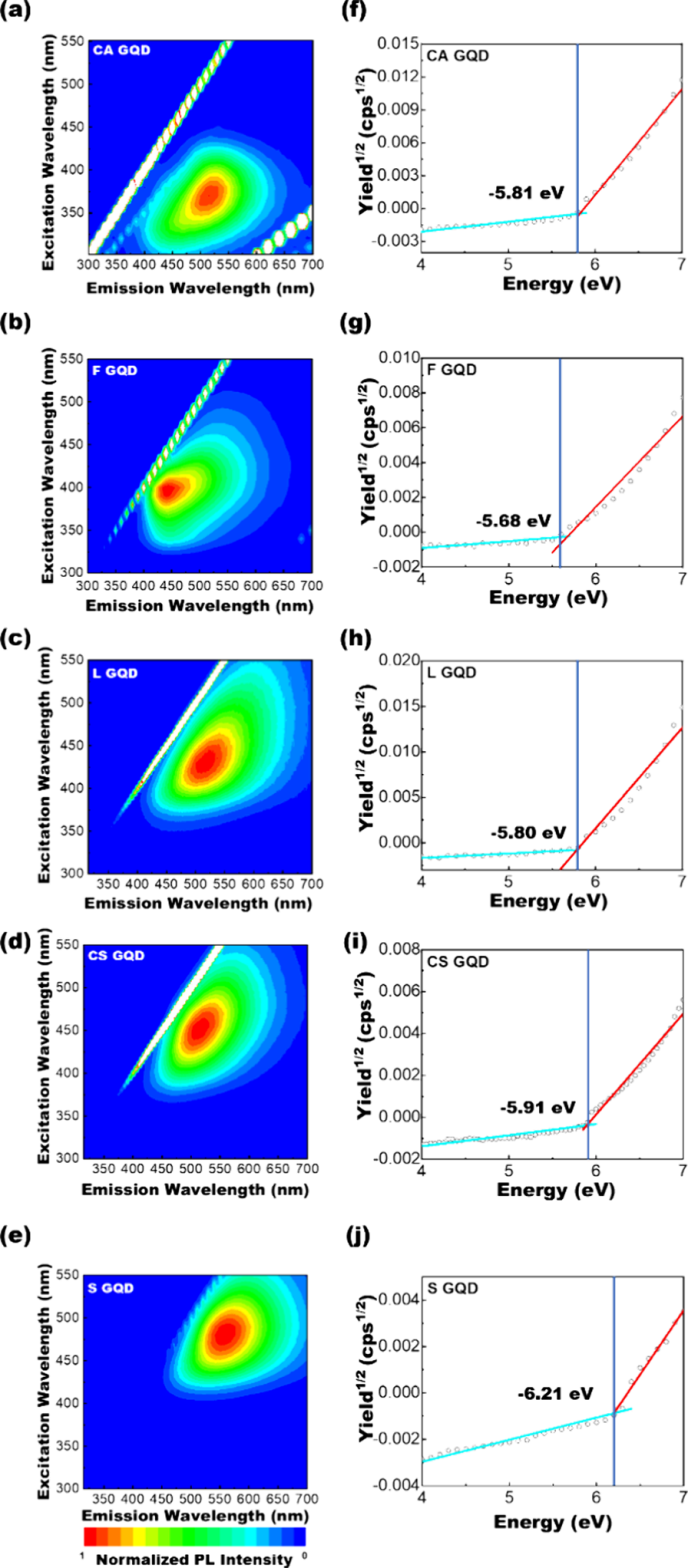
Photoluminescence spectroscopy and UPS study of plasma-synthesized GQDs. Photoluminescence (PL) maps of (a) CA-GQD, (b) F-GQD, (c) L-GQD, (d) CS-GQD, and (e) S-GQD. UPS spectra of (f) CA-GQD, (g) F-GQD, (h) L-GQD, (i) CS-GQD, and (j) S-GQD.

Figure 3a–e shows detailed PLE maps of the synthesized GQDs, and the corresponding PL spectra of synthesized GQDs under different excitations are shown in Figure S1a–e. Based on the PL data, all the GQD samples synthesized from the different bioresources showed clear PL emissions. The discernibly heightened PL intensity of CS-GQD can be ascribed to N doping, which represents electron-donating N atoms and surface functional groups. Moreover, the PL peaks for CS-GQD, L-GQD, and S-GQD were redshifted in comparison with those of F-GQD and CA-GQD, suggesting that the band gap structures of the synthesized GQDs from different bioresources can be different. The valence band (VB), conduction band (CB) levels, and band gaps (*E*_g_) of the synthesized GQDs were further estimated by ultraviolet photoelectron spectroscopy (UPS) and PL spectroscopy. The UPS spectra are shown in Figure 3f–j and indicate that the work functions of the CA-GQD, F-GQD, L-GQD, CS-GQD, and S-GQD can be estimated to be −5.81, −5.68, −5.80, −5.91, and −6.21 eV, respectively. The *E*_g_ values of the synthesized GQDs were estimated from their PL spectra and are summarized in Table 1. The details of the band gap estimation are provided in the Supporting Information. The observed reduction in *E*_g_ for CS-GQD compared to F-GQD could be attributed to the −NH_2_ group, facilitating the donation of lone pair electrons to the antibonding state.^41^

**Table 1:** Summarized *E*_g_, VB, and CB of Plasma-Synthesized GQDs

**samples**	**precursor**	***E***_**g**_**(eV)**	**VB (eV)**	**CB (eV)**
L-GQD	lignin	2.41	–5.80	–3.39
CS-GQD	chitosan	2.39	–5.91	–3.52
S-GQD	starch	2.22	–6.21	–3.99
CA-GQD	citric acid	2.39	–5.81	–3.42
F-GQD	fructose	2.78	–5.68	–2.90

**Figure 4:**
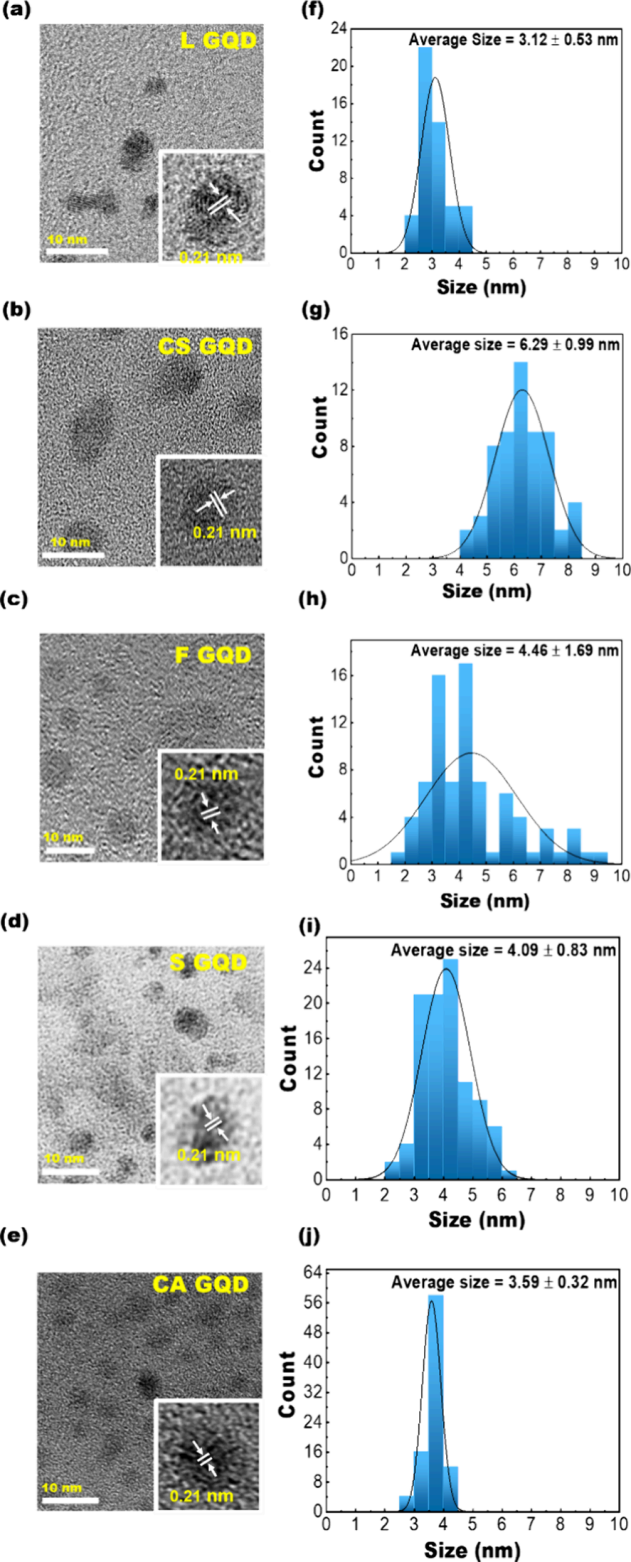
TEM study of the plasma-synthesized GQDs. TEM images of (a) L-GQD, (b) CS-GQD, (c) F-GQD, (d) S-GQD, and (e) CA-GQD. Particle size distributions of (f) L-GQD, (g) CS-GQD, (h) F-GQD, (i) S-GQD, and (j) CA-GQD.

Transmission electron microscopy (TEM) was used to study the morphology of the synthesized GQDs. TEM images of L-GQD, CS-NGQD, F-GQD, S-GQD, and CA-GQD all showed distinct particle-like nanostructure morphologies with no apparent amorphous carbon deposition, suggesting the high quality of the synthesized GQDs (Figure 4a–e). High-resolution TEM images exhibit typical graphene (100) lattice spacings of 0.21 nm (insets in Figure 4a–e). The TEM histogram of about 100 particles to a Gaussian distribution yielded average particle sizes of L-GQD, CS-GQD, F-GQD, CA-GQD, and S-GQD with the values of 3.1 ± 0.5, 6.3 ± 0.99, 4.5 ± 1.7, 3.6 ± 0.3, and 4.1 ± 0.8 nm, respectively, indicating that the size of GQDs can be controlled by the precursor structures and synthesis conditions (Figure 4f–j). Micro-Raman spectroscopy was used to study the vibrational modes of the synthesized GQDs. The Raman spectra of the synthesized GQDs exhibited D and G bands located at ∼1340–1360 and 1580–1600 cm^–1^, respectively (Figure S2), confirming the presence of graphene-related structures. A 2D band at 2780 cm^–1^ is also observed, suggesting the existence of graphene-like nanodomains in the as-produced starched-derived GQDs. The Raman spectra of all GQDs (Figure S2) reveal distinctive *I*_d_/*I*_g_ values: 1.00 for CS-GQD, 1.97 for F-GQD, 0.69 for L-GQD, 0.73 for CA-GQD, and 0.22 for S-GQD. The elevated *I*_d_/*I*_g_ values for CS-GQD and F-GQD signify heightened densities of Raman-active edge sites and reduced crystallinity in doped GQDs compared with their undoped counterparts.

**Figure 5:**
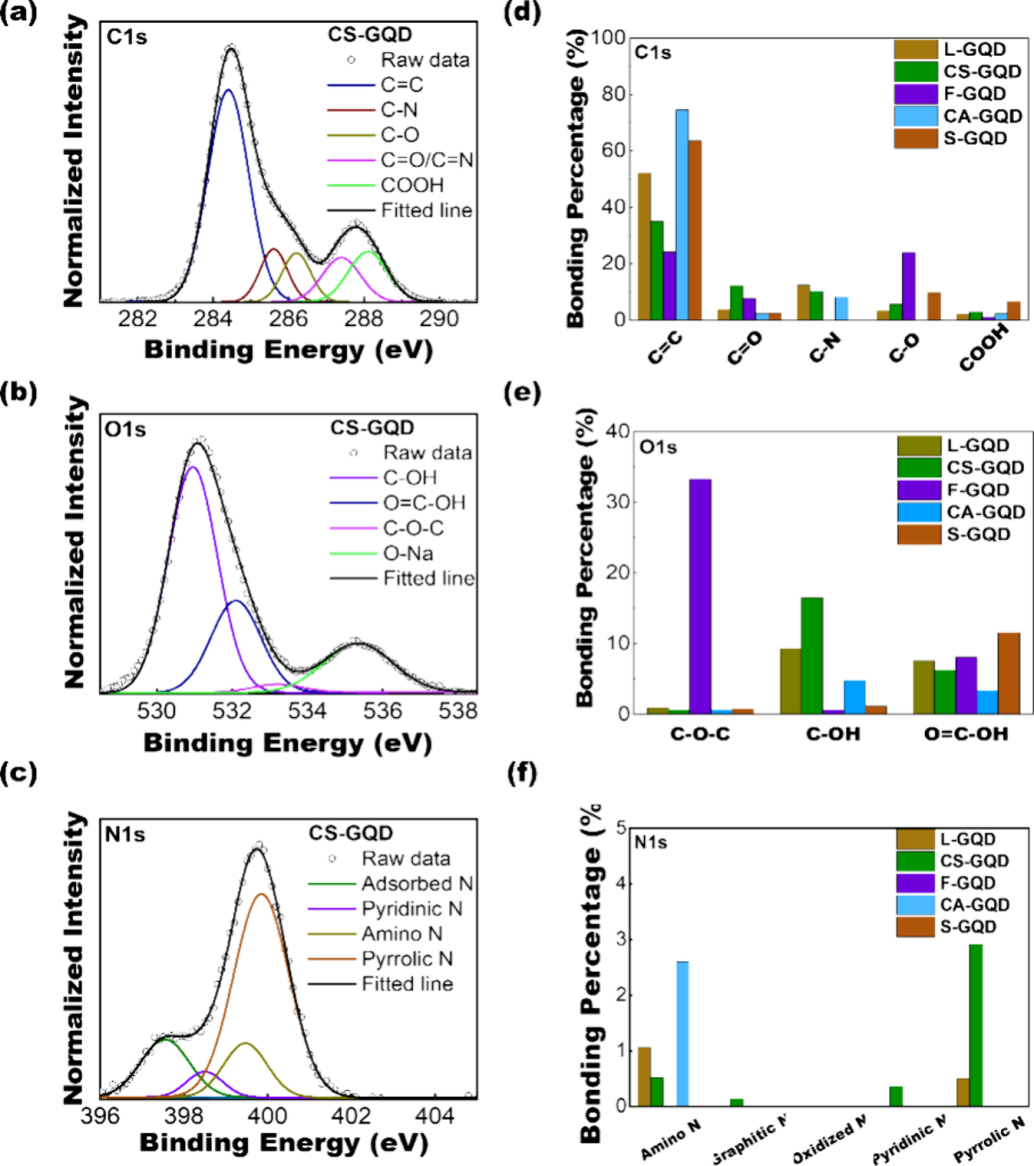
XPS analysis of plasma-synthesized GQDs. High-resolution XPS spectra of (a) C 1s, (b) O 1s, and (c) N 1s of CS-GQD. GQD bonding percentages of (d) C 1s, (e) O 1s, and (f) N 1s from the XPS data.

X-ray photoelectron spectroscopy (XPS) was used to study the chemical bond configurations of the GQDs. The survey scan reveals the elemental composition of the GQDs, showing distinctive peaks for C 1s at 283.9 eV, N 1s at 398.0 eV, S 2p at 167.9 eV, and O 1s at 530.0 eV (Figure S3). The presence of Na observed in the survey scan is ascribed to the utilization of NaOH during the synthesis of CA-GQD, F-GQD, and S-GQD, which served as an electrolyte and neutralizing agent. High-resolution XPS measurements provided detailed insights into the functionalities present in each bioresource-derived GQD (Figure 5a–c and Figures S4–S7). Taking CS-GQD as an example, deconvolution of the C 1s peak reveals five distinct peaks, corresponding to C=C (284.4 eV), C–N (285.59 eV), C–O (286.20 eV), C=O (287.31 eV), and COOH (288.09 eV).^46,47^ The O 1s peak, influenced by the NaOH electrolyte and neutralization process, exhibited C–OH (530.92 eV), COOH (532.90 eV), O=C–O (533.23 eV), and Na (535.3 eV) peaks.^46^ The N 1s peak illustrates various nitrogen functionalities, including absorbed N (397.05 eV), pyridinic N (398.50 eV), amino N (399.28 eV), pyrrolic N (399.78 eV), and graphitic N (400.87 eV).^38,40,48,49^ The bonding percentages of the different elements are depicted in Figure 5d–f, providing a detailed overview of the elemental composition of the GQDs. XPS analysis underscores the versatility of the GQDs, indicating that their functionalities can be controlled using plasma synthesis. The dopant concentrations of the synthesized GQDs are listed in Table S1. Based on the above detailed results, it is evident that photoluminescent band gap-controlled GQDs with tailored surface functionalities can be synthesized from bioresource precursors using the developed microplasma synthesis method.

### SERS Study of Plasma-Synthesized GQDs

**Figure 6:**
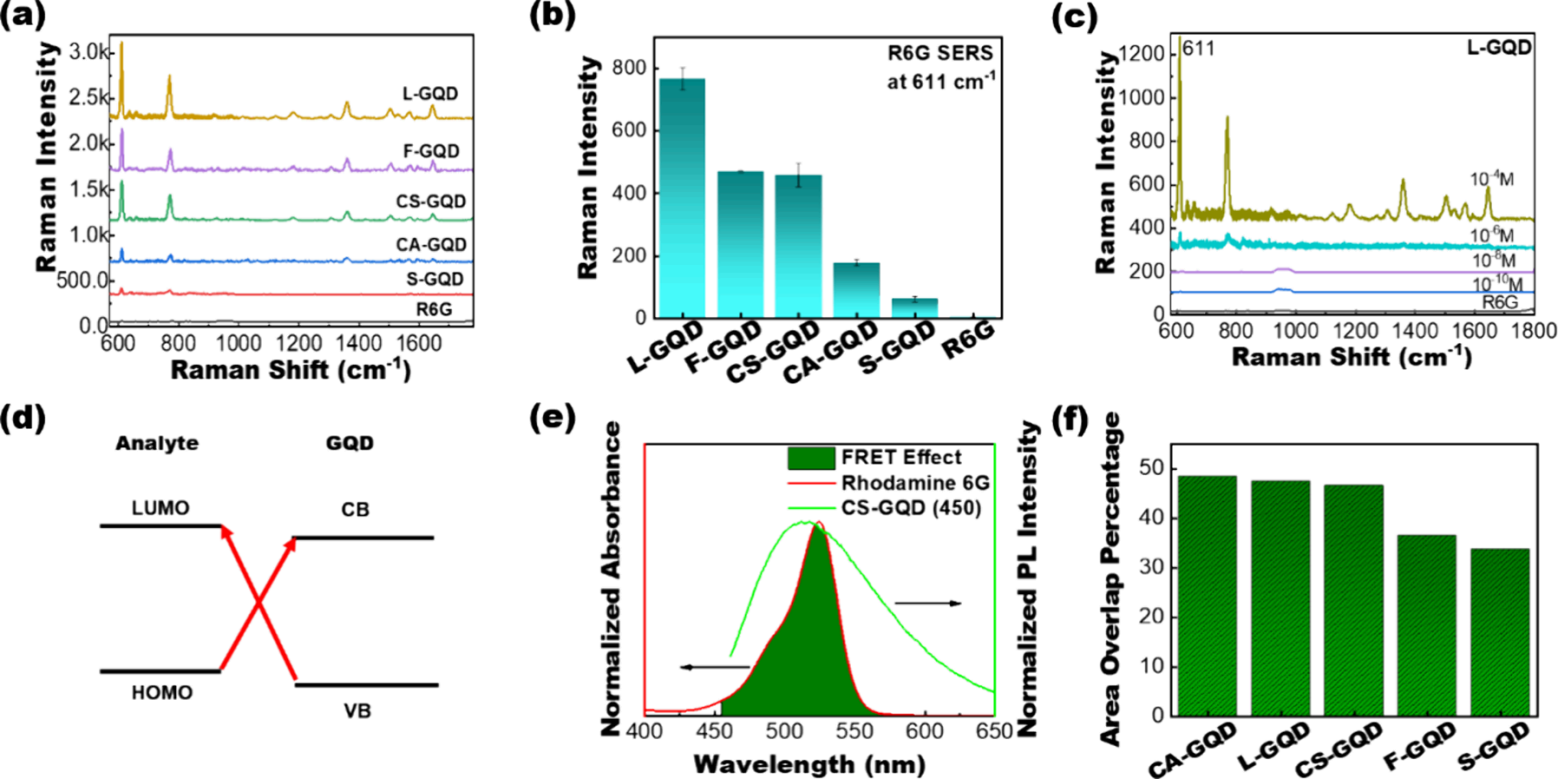
Raman scattering and FRET study of plasma-synthesized GQDs. (a) SERS spectra of R6G with different plasma-synthesized GQDs. (b) Raman intensity of R6G at 611 cm^–1^ with different plasma-synthesized GQDs. (c) SERS spectra for R6G with L-GQD. (d) Schematic of charge transfer between the GQD and analyte. (e) Normalized absorbance spectrum of RG6a and PL spectrum of CS-GQD under 450 nm excitation. The overlapped area indicates the FRET effect between the CS-GQD and R6G. (f) Overlap area percentage of FRET between GQDs synthesized from different precursors and R6G.

The SERS properties of the synthesized GQDs were studied via commercial micro-Raman spectroscopy using rhodamine 6G (R6G) as the standard Raman probe. Details of the sample preparation and Raman measurements are provided in the Experimental Section and the Supporting Information. Figure 6a shows the Raman spectra of 10^–4^ M R6G with different bioresource-driven GQDs. Enhanced R6G Raman peaks were observed at 611, 772, 1175, 1362, 1508, and 1571 cm^–1^, indicating that the synthesized GQDs exhibited SERS properties. Sulfur-doped L-GQD showed the highest enhancement among all of the test samples (Figure 6b). S and N doping in L-GQD introduces new electronic states and defects within the GQD lattice, enhancing the electronic properties and increasing the active sites for SERS by facilitating the adsorption of R6G molecules. S- and N-doped L-GQD are small compared to other GQDs. Small-size GQDs exhibit a stronger quantum confinement effect compared to larger GQDs and result in enhanced SERS performance.^50^ We further measured the Raman responses of R6G at various concentrations using the synthesized GQDs, and the results are shown in Figure 6c and Figure S8. These results indicate that the synthesized GQDs have improved SERS properties for the detection of R6G at low concentrations in the nanomolar range, which is comparable to that of conventional Ag nanoparticle-based SERS substrates.^51,52^ Two possible factors can be considered to control the SERS properties of nonmetallic materials. One is the charge transfer (CT) process between the energy levels of the GQDs and the target molecule,^53−55^ as shown in Figure 6d, and the other is the Forster resonance energy transfer (FRET) effect.^56^Figure 6e shows the absorption spectra of R6G and the PL emission spectra of the CS-GQD at 450 nm excitation. An overlap between the two spectra was observed, suggesting the occurrence of the FRET effect. The area overlap percentages between the synthesized GQDs and R6G were estimated and are shown in Figure 6f, clarifying substantial overlaps among all synthesized GQDs and R6G, especially for L-GQD and CS-GQD (Figure S9). This overlap is consistent with the observed SERS spectra. The close interaction between the energy donors (GQD) and acceptors (R6G) could be a factor in improving the Raman responses in our study.

### SERS-Based Detection of Amino Acids Using GQDs

**Figure 7:**
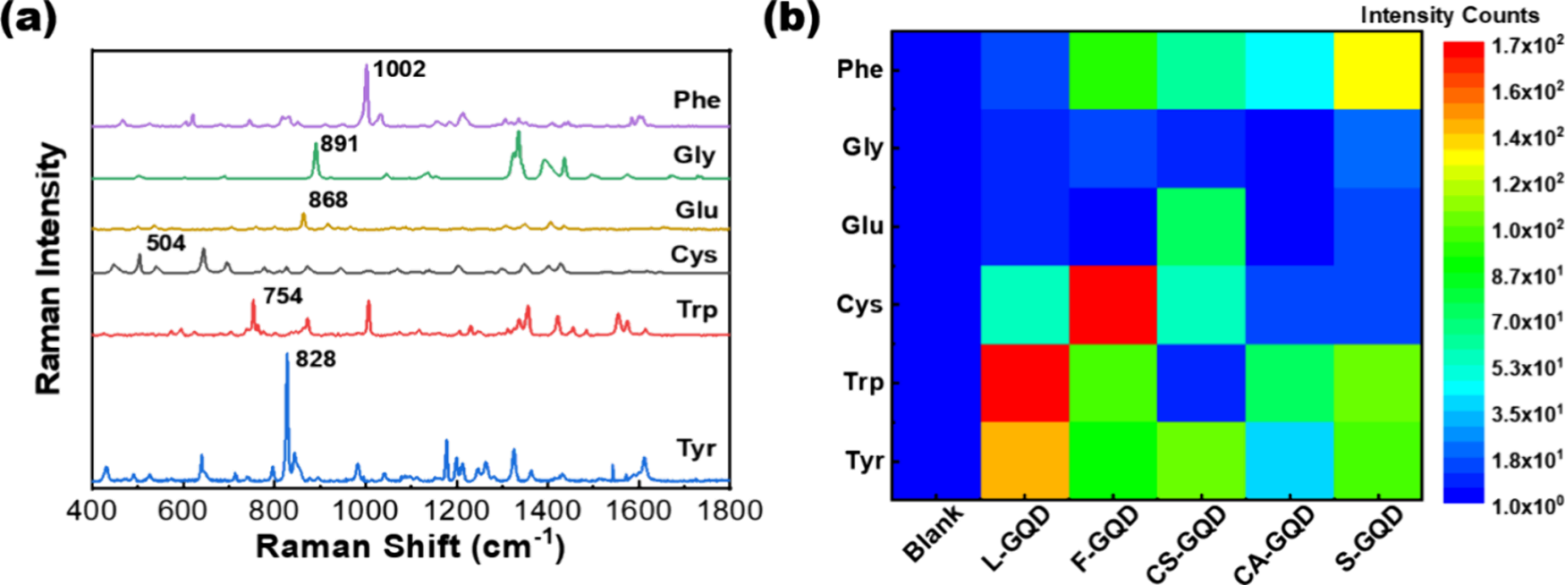
Raman spectra of amino acids and selectivity of amino acid detection for plasma-synthesized GQDs. (a) Raman spectra of the amino acid powders. The major Raman peaks of amino acids are shown. (b) Selectivity maps of amino acids with different bioresource-derived GQDs. The Raman intensities are analyzed from the major.

Six common amino acids, namely, glutamic acid (Glu), tryptophan (Trp), tyrosine (Tyr), phenylalanine (Phen), cysteine (Cys), and glycine (Gly), were selected for SERS-based detection using the synthesized GQDs. Figure 7a shows the Raman spectra of amino acid powders under ambient conditions. We noticed that tyrosine, tryptophan, cysteine, glutamic acid, glycine, and phenylalanine showed significant Raman peaks at 828, 754, 497, 868, 891, and 1002 cm^–1^, respectively, under 532 nm laser excitation, which is consistent with previous reports.^57,58^ The peak located at 520 cm^–1^ and the broad peak with a low intensity at ∼1000 cm^–1^ were attributed to the Si wafer used as the substrate. Detailed Raman peak assignments of the tested amino acids are provided in the Supporting Information. We further measured the SERS responses of six amino acids' solution (10^–3^ M concentration) dried on the synthesized GQDs. Figure 7b shows a 2D map of the SERS responses of the six amino acids with differently synthesized GQDs. The intensity is the Raman intensity of amino acids on different GQDs. Interestingly, the synthesized L-GQD exhibited a significant SERS enhancement toward Trp, whereas the F-GQD exhibited a pronounced SERS enhancement for Cys. Moreover, the CS-GQD exhibited good selectivity for Glu, and the S-GQD showed good selectivity for Phen, suggesting that selective interactions occurred between the tested amino acids and GQDs. GQDs exhibit unique selectivity properties for amino acids in SERS detection. Despite the enhancement in properties that doping can bring, it has been observed that doped GQDs might not always provide the desired selectivity to detect a specific amino acid in SERS applications. This suggests that the intrinsic characteristics of undoped GQDs such as their surface chemistry and electronic structure can play a critical role in their selectivity, potentially outperforming their doped counterparts in certain scenarios. The presence of diverse surface functional groups on the GQDs, introduced by different precursors, ensures significant interaction and selective sensing of amino acids by facilitating effective charge transfer.^59−62^

**Figure 8:**
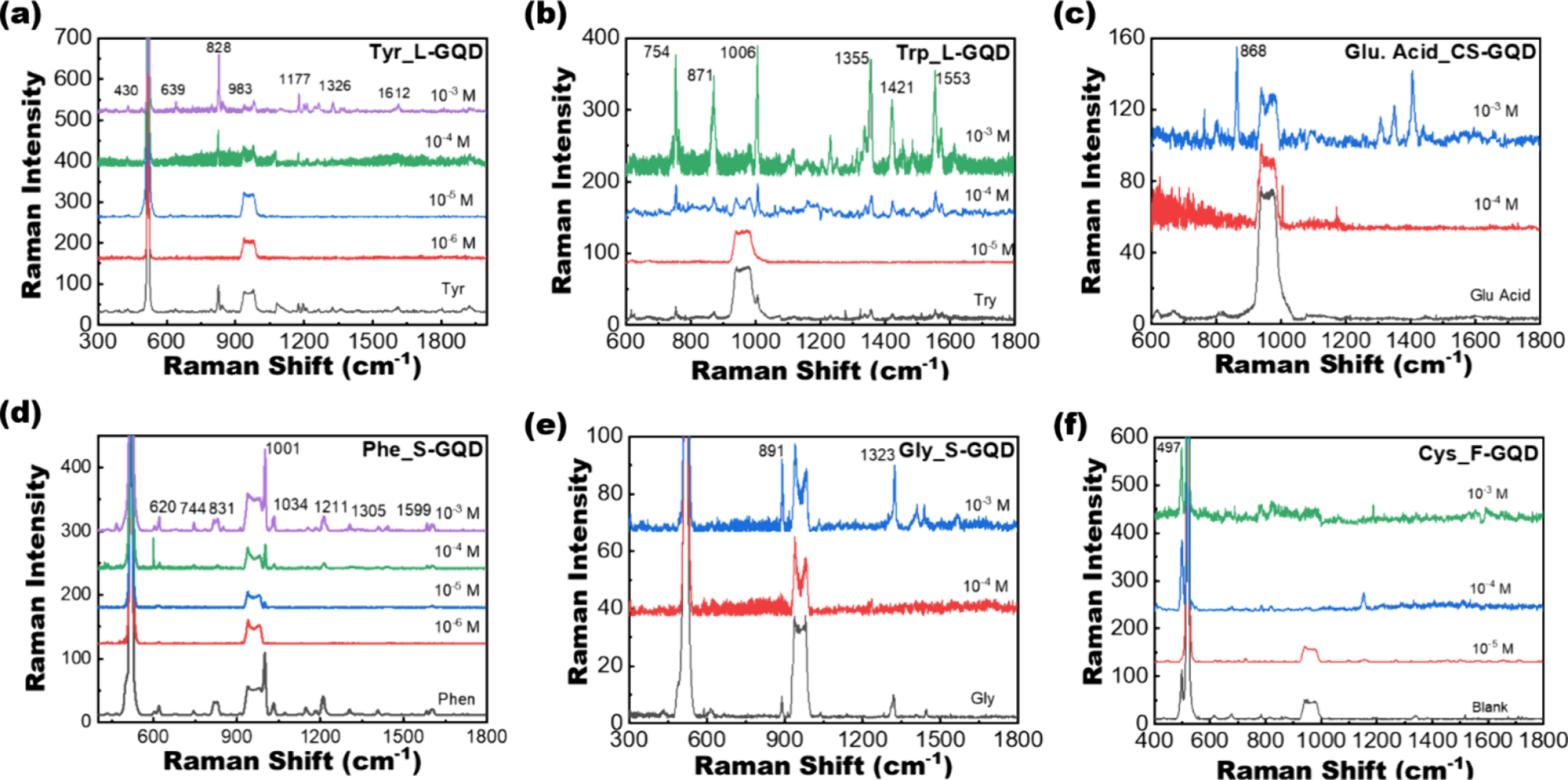
SERS-based detection of amino acids with different plasma-synthesized GQDs. SERS spectra of (a) Tyr with L-GQD, (b) Trp with L-GQD, (c) Glu acid with CS-GQD, (d) Phen with S-GQD, (e) Gly with S-GQD, and (f) Cys with F-GQD.

Based on the selectivity results shown in Figure 7b, we measured the SERS responses of the target amino acids with the corresponding GQDs. Figure 8 demonstrates the systematic enhancement of characteristic Raman peaks of various amino acids with bioresource-derived GQDs within the concentration range of 10^–3^–10^–6^ M. Each amino acid displays distinct SERS spectra, with Tyr exhibiting a significant peak at 828 cm^–1^ (Figure 8a), Trp at 754 cm^–1^ (Figure 8b), Glu acid at 868 cm^–1^ (Figure 8c), Phen at 1001 cm^–1^ (Figure 8d), Gly at 891 cm^–1^ (Figure 8e), and Cys at 497 cm^–1^ (Figure 8f). Among these spectra, Glu and Gly do not demonstrate a detection limit below 10^–3^ M. Various factors contribute to the SERS enhancement of these amino acids including their molecular structure, chemical reactivity, and specific interactions formed with the surface functional groups of the GQDs. Although Gly is thermally unstable and may be easily decomposed during Raman measurements, the solvation effect of amino acids due to the structural arrangement of water molecules around the amino acids can be another factor limiting the SERS response.^63^ Gly and Glu amino acids represent aliphatic side chains and acidic-amide side chain amino acids, respectively. Therefore, the lower intensity observed in the SERS spectra of Gly and Glu acid compared to the higher intensity signals exhibited by other amino acids suggests that bioresource-derived GQDs exhibit a higher degree of interaction with aromatic side chain amino acids. Conversely, aromatic side chain-containing amino acids, such as Tyr, Trp, and Phen, which feature aromatic rings in their structures, display heightened SERS signals because of their enhanced interaction with the GQDs. Previous work has shown that GQDs interact more with aromatic amino acids through π–π* stacking, resulting in enhanced fluorescence, but there is no π–π* stacking with nonaromatic amino acids, which leads to fluorescence quenching.^64^ Furthermore, the significant SERS enhancement observed for Cys can be attributed to the presence of highly oxygen-containing functional groups on the GQD surfaces. Cys, which is known for its high reactivity among amino acids, likely interacts with these oxygen-containing surface functional groups, leading to intensified SERS signals. Nonetheless, further exploration of these factors is warranted to understand and optimize the SERS performance of biomolecule-GQD systems.^65^ Overall, our study achieved a remarkable detection limit of 10^–5^ M, demonstrating the potential of bioresource-derived GQDs as nonmetal SERS nanoprobes for amino acid detection. Table S3 shows the amino acid concentrations in a healthy adult human plasma. The enhancement factors (EF) for amino acids are 9.84, 24.21, 29.00, 9.47, 4.60, and 14.29 for Tyr, Trp, Glu acid, Phen, Gly, and Cys, respectively. The details of calculation of EF using formula S1 are shown in the Supporting Information.

### Theoretical Calculation

The band gap structures, photoluminescence properties, and surface states, including dopants and functional groups, may play a role in the observed SERS response of amino acids. Previous studies have suggested that CT-induced SERS enhancement is largely dependent on the coupling between the conduction and valence bands of SERS-active materials with the highest occupied molecular orbital (HOMO) and the lowest unoccupied molecular orbital (LUMO) of the absorbed target molecule. To understand the CT-induced SERS effect, we studied the band gap structures of the synthesized GQDs. Density functional theory (DFT) calculations were also performed to obtain the energy levels of the HOMO and LUMO of the amino acids (details in the Supporting Information). In general, two major CT paths occur during Raman scattering. CT1 represents the excitation electrons from the HOMO of the target molecules to the CB of the GQDs, whereas CT2 represents the excited electrons from the VB of the GQDs to the LUMO of the target molecules. As shown in Figure S10, we observed two distinct pathways for CT between the GQDs and amino acids, denoted as CT1 and CT2. The CT1 energies between the GQDs and specific amino acids were found to be closer than that of path CT2 to the incident laser energy used (2.33 eV, 532 nm excitation). When the CT paths between the GQDs and the target molecule closely align with the incident excitation energy (2.33 eV, 532 nm laser excitation in this study), an enhanced SERS response can be promoted. Based on the above results, it is evident that the energy-level difference for CT1 is significantly lower than that for CT2. Consequently, CT1 emerged as a more favorable pathway for charge transfer, indicating its potential role in facilitating SERS enhancement.

To further study the occurrence of CT between the GQDs and the amino acids, the energy levels of the GQDs adsorbed with the target amino acids were calculated by using DFT calculations. The details are provided in the Supporting Information (Figures S11–S13). The red and green colors in the HOMO–LUMO represent the positive and negative orbitals, respectively. Table S2 presents the HOMO–LUMO energy levels for both the GQD and amino acids on the GQD substrate. This table illustrates the shift in the energy levels after the adsorption of amino acids on the GQDs. Slight changes in the energy levels and *E*_g_ confirmed the occurrence of CT between the GQDs and the amino acids. In the case of the GQDs with Gly and Glu, there was little or no change at every level and *E*_g_, explaining the low SERS enhancement. Conversely, Tyr and Phen showed significant CT, leading to a high SERS enhancement and low LoD. Our research suggests that tuning the band gaps of GQDs can be an effective approach for improving the SERS properties of graphene-based materials for SERS-based applications. To investigate charge transfer between GQDs and amino acids, we employed Raman spectroscopy and UV–vis spectroscopy.^62,66−69^Figure S14 shows the Raman spectroscopy study to directly demonstrate the charge transfer mechanism between the GQD and the target amino acid molecule. Raman results obtained for amino acids with the GQD substrate and without the GQD substrate reveal a noticeable peak shift of the analyte upon contact with the GQD SERS substrate, indicating the occurrence of a charge transfer mechanism. These shifts in Raman results provide clear information about the interaction between GQDs and the target molecule. Furthermore, Figure S15 shows the UV–vis absorbance spectra of GQDs with and without their selective amino acids. The spectra reveal shifts or changes in absorption bands, indicative of electronic transitions associated with the charge transfer phenomenon.

## Conclusions

The development of rapid, sensitive, and selective chemical and biomolecule detection is important not only for fundamental studies but also for the establishment of a healthy society. However, conventional detection methods have been hampered by the difficulties of low sensitivity, long sampling and detection times, and expensive operation and instruments. In this study, we report the plasma engineering of bioresource-derived GQDs as SERS-active materials for the rapid and sensitive detection of amino acids. Surface-functionalized GQDs with tuned structures and band gaps were synthesized from biocompatible earth-abundant bioresources using reactive microplasmas under ambient conditions. The SERS properties of the synthesized GQDs were tuned by engineering their band gaps of synthesized GQDs. The plasma-synthesized metal-free GQDs with surface functionalities showed enhanced SERS properties for rapid and sensitive amino acid detection with low detection limits of 10^–5^ M for tyrosine and phenylalanine. Theoretical calculations suggest that charge transfer between GQDs and amino acids could be a factor in enhancing the Raman scattering of GQDs. Our research not only provides a strategy to produce nanographene-based materials in a simple and environmentally friendly manner but also provides insight for the development of advanced sensing platforms for emerging materials science, chemistry, nanotechnology, and biomedicine.

## Experimental Section

### Materials and Chemicals

Chitosan (50–190 kDa), lignin alkali (MW ∼ 10 kDa), starch, fructose (≥99%), and sodium hydroxide (≥98%) were purchased from Sigma-Aldrich. Citric acid (99.5%) was purchased from Acros Organics (USA). Ammonium hydroxide (28.0–30.0%) was obtained from J.T. Baker. Platinum foil (99.95%, 20 mm × 20 mm × 0.1 mm) was purchased from GuV Team International. l-Phenylalanine (99%), l-tyrosine (99%), and l-glutamic acid (99%) were purchased from Alfa Aesar. Glycine (>99%), l-lysine (>98%), l-tryptophan (>99%), and cysteine (>98%) were obtained from Acros Organics. The amino acids were used without L for further discussion.

### Plasma Synthesis of GQDs

Briefly, for the preparation of lignin GQDs, a precursor solution for lignin GQDs was prepared by adding 3.75 mL of lignin solution (2.4 × 10^–4^ M), 1 mL of NH_4_OH (1 M) solution, and 5.25 mL of deionized (DI) water. Ten mL of the solution was then used for microplasma treatment under a plasma current of 5 mA for a 1 h reaction time. To prepare the chitosan GQDs, 300 mg of chitosan was dissolved in 19.6 mL of DI water and stirred for 5 min. Acetic acid (5 M, 0.4 mL) was added after mixing and stirred again for 5 min. The solution was thoroughly mixed by adding 20 mL of DI water. A 10 mL solution was then used for microplasma treatment under a plasma current of 9.6 mA for a 1 h reaction time. The fructose GQD precursor was prepared by adding fructose solution (0.067 M), NaOH (0.04 M), and DI water. A 10 mL solution was then used for microplasma treatment under a plasma current of 9.6 mA for a 1 h reaction time. The same procedure was followed for the synthesis of the GQDs using CA and starch. After plasma synthesis of the GQD, fructose and citric acid GQDs were purified by dialysis against DI water for 24 h. The solution was adjusted to the acidic conditions of lignin by the addition of HCl (1 M). After addition of HCl, the residue was removed by centrifugation. The obtained solution was dialyzed for 24 h after neutralization using NaOH (1 M). To purify the S-GQD, ethanol was used to precipitate unreacted starch from the GQD solution. The residue was removed by centrifugation. The obtained solution was dialyzed after the removal of ethanol using a rotary evaporator (∼10 hPa; Eyela vacuum controller, Japan). For CS-NGQD, the initial pH was adjusted to 7 or 8 by adding NaOH (1 M). To precipitate the unreacted chitosan, acetone was added to the CS-NGQD solution at a ratio of 2:1. The precipitate solution was removed by centrifugation, and the solution was dialyzed after acetone was removed using a rotary evaporator. After dialysis, a rotary evaporator was used to remove water and obtain a fine GQD powder.

### Characterization

PL measurements of all bioresource-GQDs were performed using a Horiba fluorophore spectrometer at room temperature. All the GQD absorbance spectra were captured using a JASCO V676 absorption spectrophotometer fitted with two quartz cuvettes with a path length of 1 cm. Deionized water was used as the baseline correction reference for each measurement. XPS data were obtained using an ESCALAB Xi+ (Thermo Fisher Scientific, United Kingdom) with monochromatic Al Kα X-ray radiation as the source gun, a pass energy of 150.0 eV, and a beam size of 650 μm. TEM measurements were performed using a field-emission gun TEM (FEI Tecnai G2 F-20 S-TWIN) at an acceleration voltage of 200 kV. Samples for TEM were prepared by solution-drying colloidal all-bioresource-purified GQD solutions on carbon-coated copper grids of 400 mesh. Raman spectra for bioresource-derived GQDs and SER spectra were recorded at room temperature by using a micro-Raman JASCO 5100 spectrometer (laser excitation at 532 nm).

### SERS Measurement

A GQD solution of 0.5 mg mL^–1^ was prepared in DI water. Purified GQDs were further used without any surface coating. The different surface functionalities present on GQDs were due to different precursors. Silicon wafers were used to detect the presence of R6G and other amino acids. To prepare the SERS sample, 50 μL of the GQD solution was dropped onto a Si wafer and dried for ∼40–60 min at 45–50 °C. The substrate was then soaked in a Raman probe (R6G and amino acids) solution for 5 min and dried at 35–40 °C for ∼1 h. It is plausible that some amino acid molecules may interact with GQD while some amino acids molecules form aggregates upon drying. The Raman spectra were obtained after drying the sample. The SERS spectra were obtained at 532 nm (2.33 eV) with an integration time of 30 s. To confirm the accuracy of the results, the intensity of the significant peak of each analyte (R6G and amino acids) was calculated on average from 12 measured sites on the substrate at different locations on the substrate.

### Theoretical Calculation

The DFT calculations were performed by using the Gaussian/g16 program. Accurate data prediction was achieved by combining the Becke exchange functional (Becke’s three-parameter) and Hartree–Fock exchange. The 6-311G split-valence of the B3LYP hybrid functional, which was derived from LYP (Lee, Yang, and Parr), describes inner-shell orbitals using six Gaussian functions and a split-valence set of four Gaussians for valence orbitals with subsets 3, 1, and 1.^70^ Various software tools, including ChemDraw, Chem3D, GaussView 6, and Avogadro, were used for structure generation and subsequent analysis. To explore the interaction between the amino acids and GQDs, a surface-on model was used. The surface structures of the GQDs were designed according to the XPS data by adding functional groups with elemental doping information.

## Supporting Information Available

Additional PL, Raman, DFT, and XPS results (PDF)
