# Supplementary material for: Tuning Nanographene-Enhanced Raman Scattering for Rapid Label-Free Detection of Amino Acids
Source: ACS Appl Mater Interfaces. 2024 Sep 24;16(40):54378–89. doi: 10.1021/acsami.4c08298 (PMC11472263; doi:10.1021/acsami.4c08298)
Supplement: Supplementary file 1 — am4c08298_si_001.pdf [file am4c08298_si_001.pdf]

Supporting Information

Tuning Nanographene-Enhanced Raman Scattering for Rapid Label-Free Detection of Amino Acids

Neha Sharma[1], Muhammad Hussnain Akmal[1], Ryoto Yura[2], Seyyed Mojtaba Mousavi[1], Darwin Kurniawan[1], Yoshiyuki Nonoguchi[2] and Wei-Hung Chiang[1,3,4]*

[1]Department of Chemical Engineering, National Taiwan University of Science and Technology, Taipei, 10607 Taiwan

[2]Faculty of Materials Science and Engineering, Kyoto Institute of Technology, Kyoto 606-8585, Japan

[3]Sustainable Electrochemical Energy Development (SEED) Center, National Taiwan University of Science and Technology, Taipei City 10607, Taiwan

[4]Advanced Manufacturing Research Center, National Taiwan University of Science and Technology, Taipei City 10607, Taiwan

*Corresponding author: E-mail address: whchiang@mail.ntust.edu.tw (W.H.C)

S1. Raman measurement of amino acids

Tyrosine showed significant Raman peaks at 430 cm^{-1}, 639 cm^{-1} (benzene ring deformation), 828 cm^{-1} (out-of-plane deformation), 983 cm^{-1} (Cα-Cβ, C−H wag), 1177 cm^{-1} (C−C−H (benzene ring) stretching, C−H (benzene ring bending), 1326 cm^{-1}, and 1612 cm^{-1}.[1, 2] In addition, tryptophan Raman peaks appear at 754 cm^{-1} (benzene ring, pyrrole ring breathing), 871 cm^{-1} (H-bending on pyrrole ring), 1006 cm^{-1} (ring breathing), 1354 cm^{-1}, 1421 cm^{-1}, and 1553 cm^{-1} (indole ring stretching).[1, 3] Significant Raman peaks for cysteine appear at only 497 cm^{-1} (stretching of the S-S bond in the dialkyl disulfides)[4] whereas a strong Raman band for glutamic acid is located at 865 cm^{-1} (COOH deformation vibration), 1349 cm^{-1}, and 1407 cm^{-1} (symmetric vibration of COO^-).[4, 5] Moreover, Raman peaks for glycine appeared at 891 cm^{-1} and 1323 cm^{-1} [4], and Raman peaks for phenylalanine were observed at 620 cm^{-1} (benzene ring deformation, benzene ring breathing), 744 cm^{-1} (COO− deformation, benzene ring breathing), 817 cm^{-1} (C−C stretching, Cα-N stretching), 1002 cm^{-1} (C−C benzene ring stretching, C−C−C bending), 1032 cm^{-1} (in-plane benzene ring C−H bending, 1210 cm^{-1} (benzene ring C-C stretching, C−C−H stretching, NH_2 bending, Cα-N stretching, NH_2 bending, $C-CH_2$ stretching), 1305 cm^{-1} (C−H, C−C vibrations), and 1599 cm^{-1} (R stretching, COO− asymmetric stretching).[6] A peak located at 520 cm^{-1} and a broad peak with low intensity at ~1000 cm^{-1} were attributed to the Si wafer, which was used as the substrate for sample preparation.

S1: PL spectra of all five GQDs including, L-GQD, CS-GQD, F-GQD, CA-GQD and S-GQD

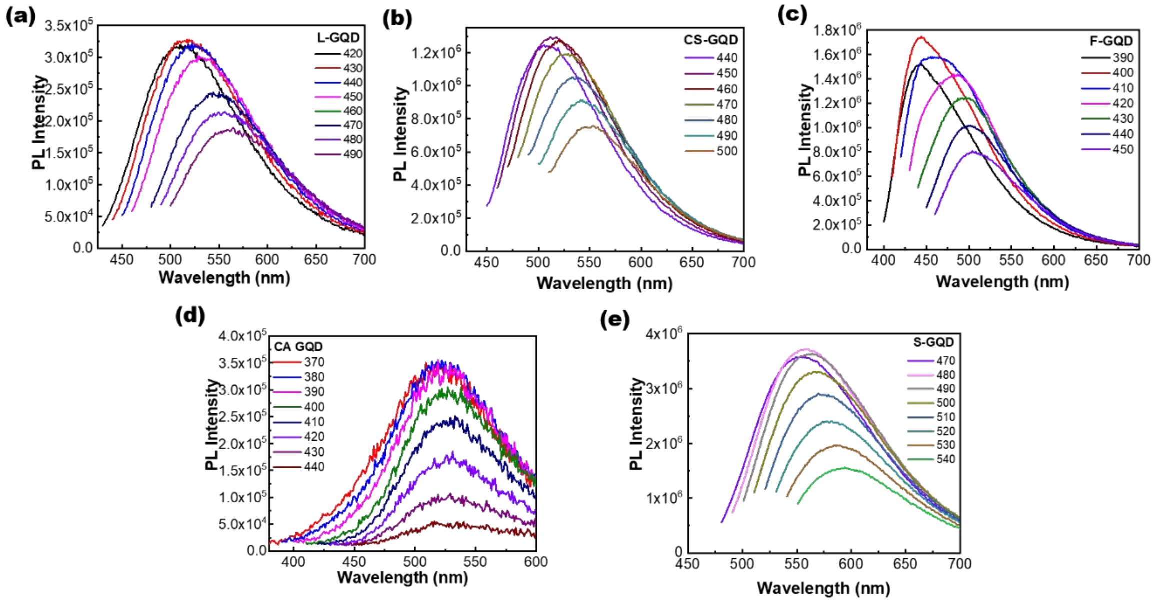

Figure S1. PL spectra of (a) L-GQD, (b) CS-GQD, (c) F-GQD, (d) CA-GQD, and (e) S-GQD.

S2: Raman Spectra of all five GQDs including, S-GQD, CA-GQD, L-GQD, F-GQD and CS-GQD

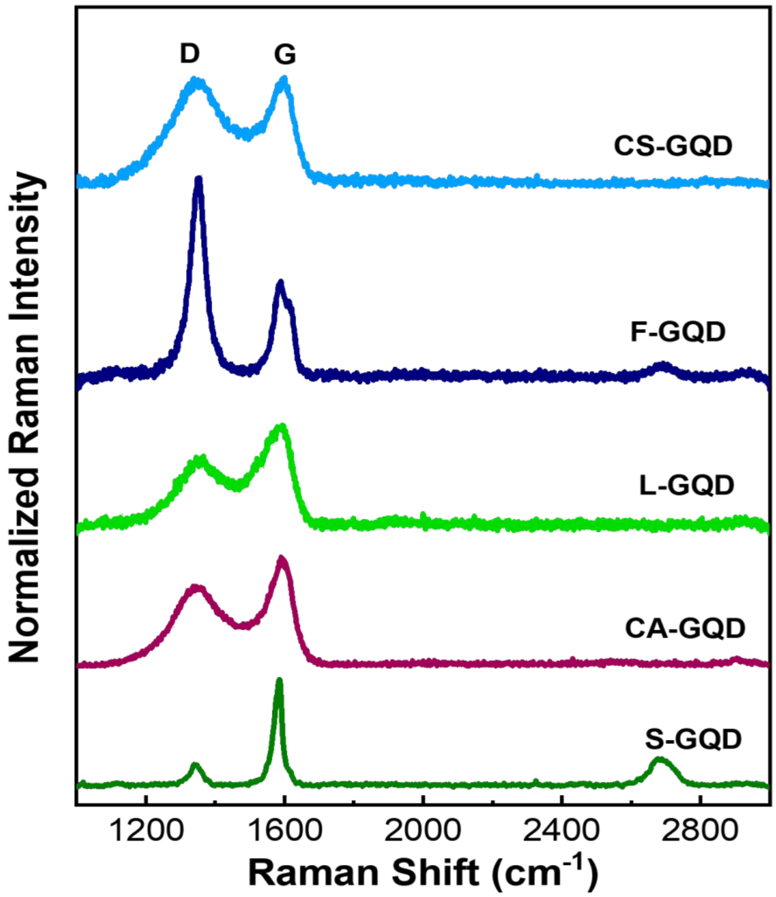

Figure S2. Raman Spectra of synthesized GQDs.

S3: Full survey XPS analysis of all five GQDs including, S-GQD, CA-GQD, L-GQD, F-GQD and CS-GQD

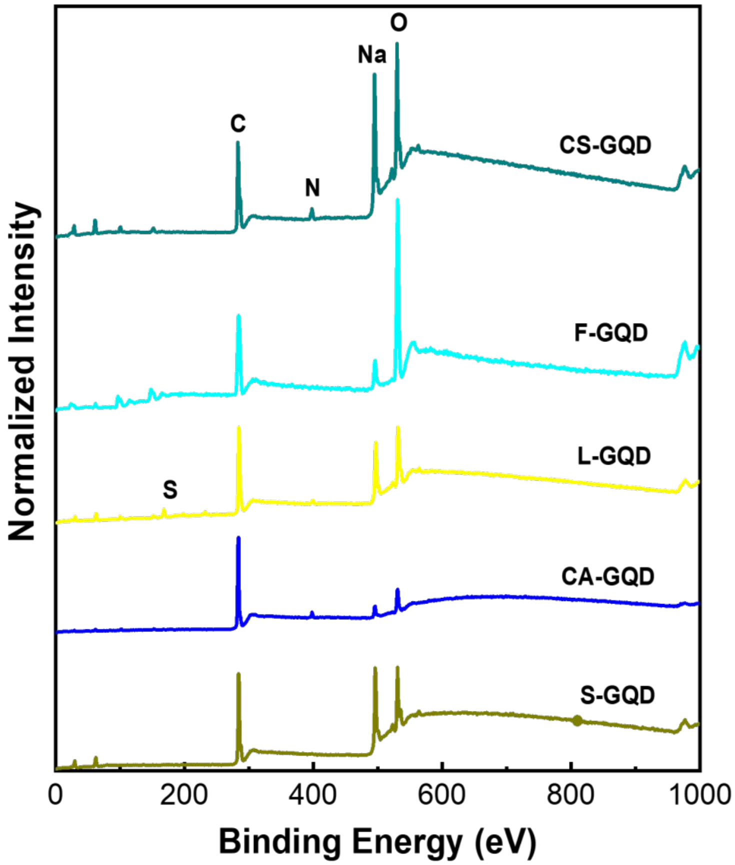

Figure S3. XPS full survey scans of synthesized GQDs

S4: High-resolution XPS measurement of L-GQD.

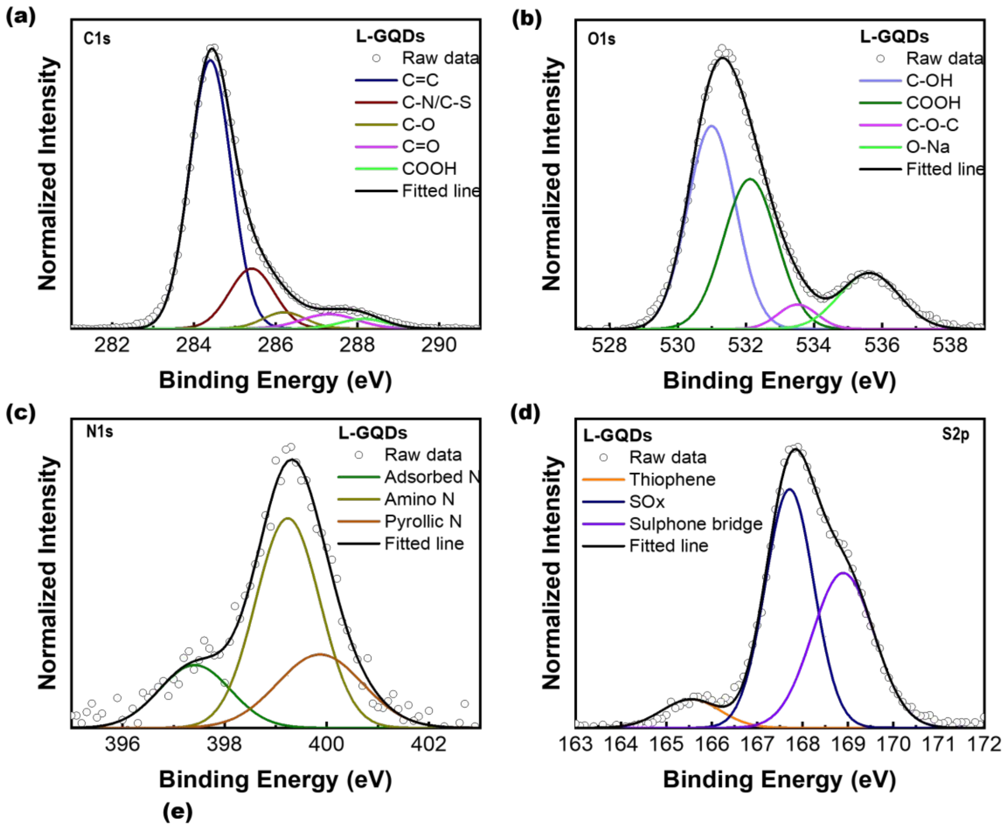

Figure S4. High-resolution XPS measurements of (a) C1s, (b) O1s, (c) N1s, and (d) S2p for L-GQD.

S5: High-resolution XPS measurement of CA-GQD.

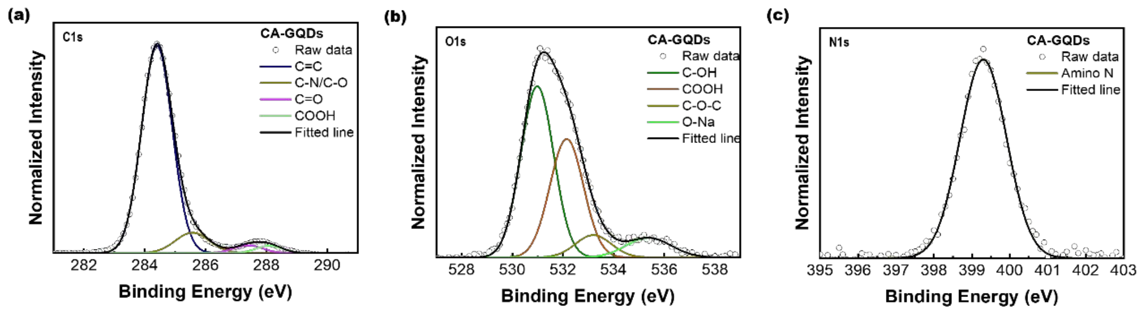

Figure S5. High-resolution XPS measurements of (a) C1s, (b) O1s, (c) N1s, for CA-GQD.

S6: High-resolution XPS measurement of F-GQD.

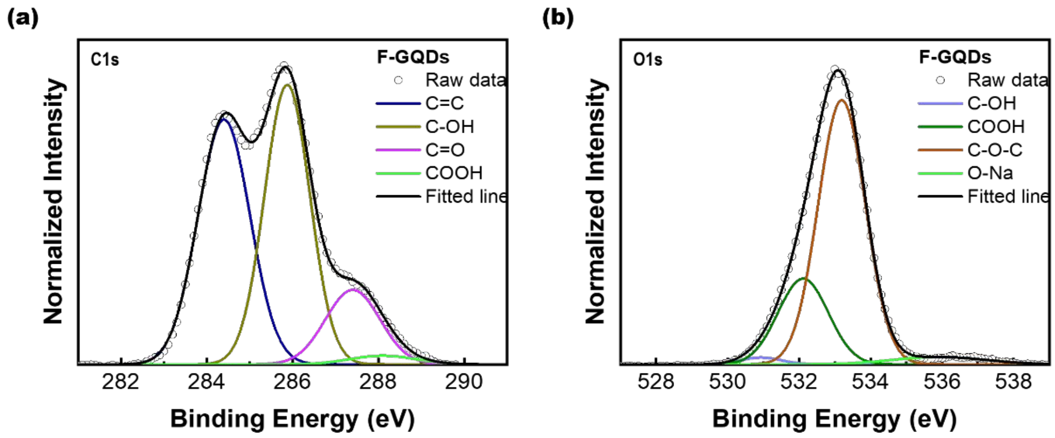

Figure S6. High-resolution XPS measurements of (a) C1s, (b) O1s for F-GQD.

S7: High-resolution XPS measurement of S-GQD.

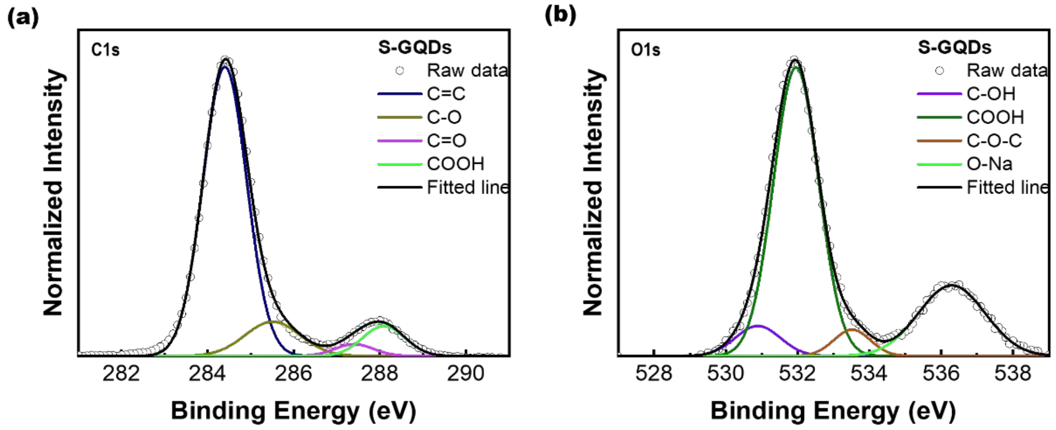

Figure S7. High-resolution XPS measurements of (a) C1s, (b) O1s for S-GQD.

S8: SERS spectra of R6G solutions using (a) CS-GQD, (b) F-GQD, (c) CA-GQD, and (d) S-GQD.

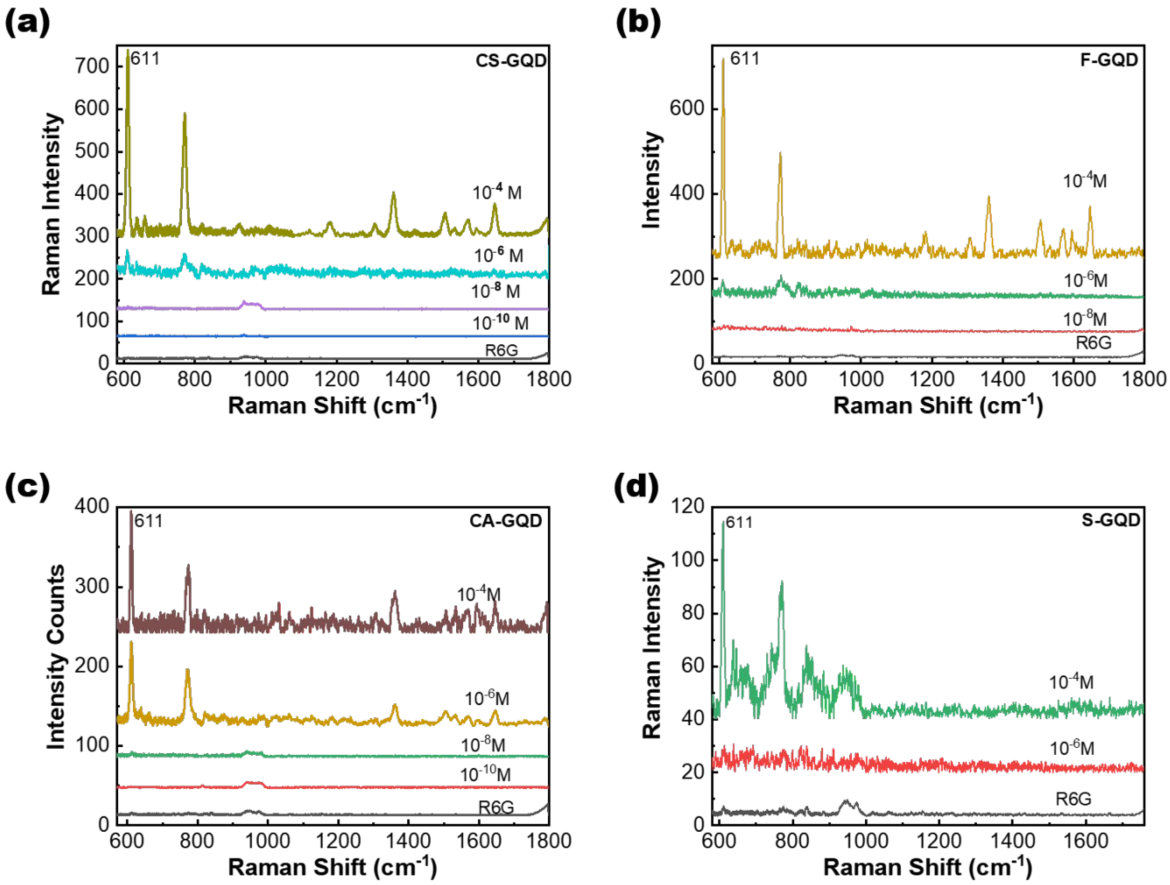

Figure S8. SERS spectra of R6G solutions using (a) CS-GQD, (b) F-GQD, (c) CA-GQD, and (d) S-GQD.

S9: FRET effect between GQD and R6G.

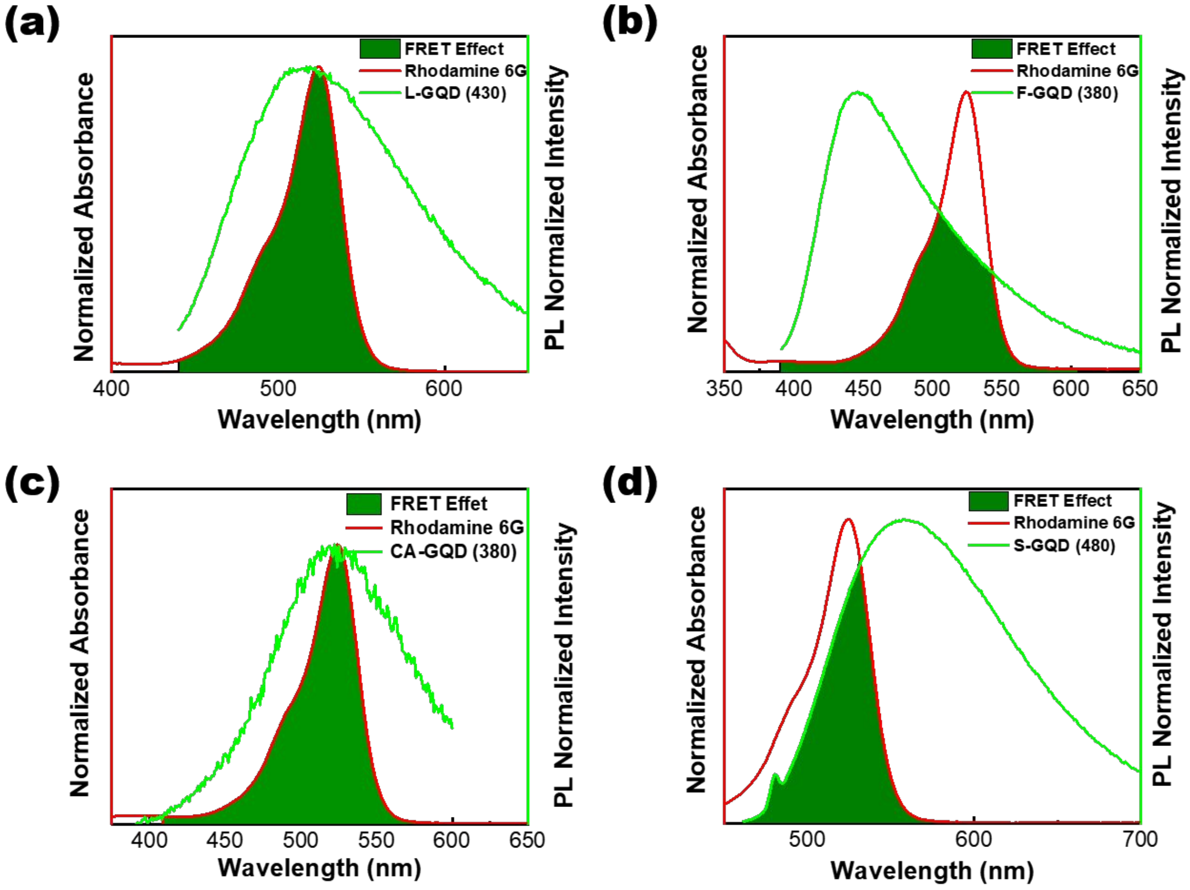

Figure S9. **Normalized absorbance spectrum of RG6 and PL spectrum of different bioresource GQDs under excitation.** The overlapped area indicates the FRET effect between (a) L-GQD and R6G, (b) F-GQD and R6G, (c) CA-GQD and R6G, and (d) S-GQD and R6G.

Table S1. Atomic percentage of elements containing in the synthesized GQDs.

Sample	Atomic percentage (at %)			
	C	O	N	S
L-GQD	74.20	21.20	1.90	2.80
CS-NGQD	66.46	28.88	4.66	-
CA-GQD	87.70	9.70	2.60	-
S-GQD	82.10	17.90	-	-
F-GQD	56.90	43.10	-	-

Table S2. calculated the HOMO, LUMO, and Energy Gap (Eg) of all GQDs and their selective amino acids

Samples	VB	CB	Eg (eV)
F-GQD	-4.35	-2.78	1.57
Cys-F-GQD	-4.57	-2.99	1.58
CS-GQD	-4.52	-2.50	2.02
GA-CS-GQD	-4.54	-2.59	1.95
S-GQD	-4.49	-2.91	1.58
Gly-S-GQD	-4.50	-2.91	1.59
Phen-S-GQD	-4.60	-3.02	1.58
L-GQD	-4.84	-3.89	0.95
Tyro-L-GQD	-4.84	-3.95	0.89
Tryp-L-GQD	-4.84	-3.86	0.98

Table S3 Amino acid concentration in normal adults reported by Jackson Alan A. et al.[a]

Amino Acid	Healthy adult blood plasma Concentration (μM)
Tryptophan	46 ± 5.4
Phenylalanine	53 ± 2.5
Glutamic Acid	167 ± 41.0
Glycine	198 ± 18.0
Cysteine	13 ± 2.4
Tyrosine	50 ± 4.8

a ; Jackson Alan A. et al./Am J Physiol Gastrointest Liver Physiol 281: G1179–G1187, 2001[7]

S10: **Possible paths of charge transfer between amino acid molecules and GQDs**

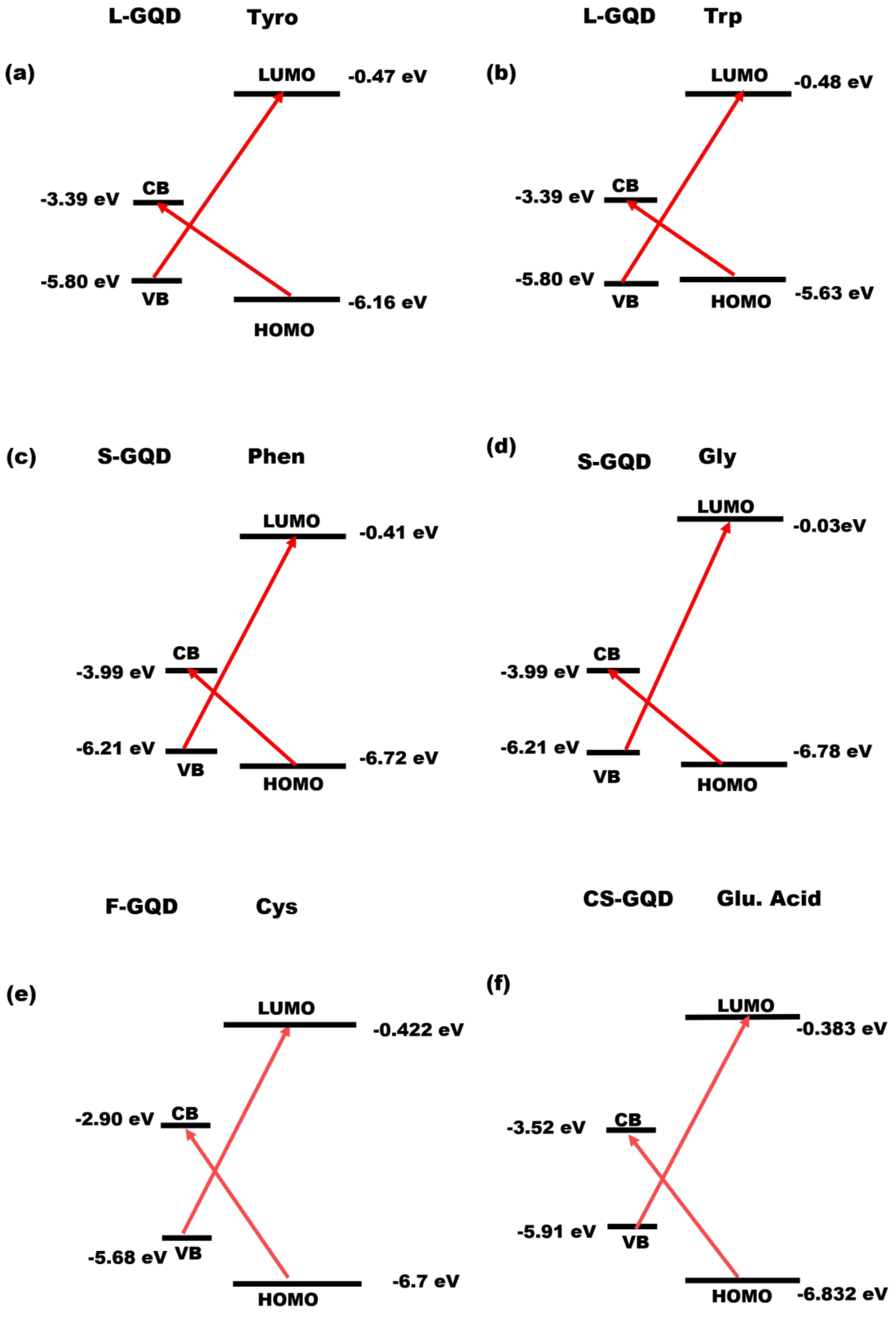

Figure S10. The illustration of possible paths of charge transfer between the valence band of the GQDs to the LUMO of amino acids and the HOMO of amino acids to the conduction band of the GQDs. CT1 represents the excitation electrons from the HOMO of the target molecules to the CB of the GQDs, whereas CT2 indicates the excited electrons from the VB of the GQDs to the LUMO of the target molecules. Possible charge transfer paths for (a) L-GQD and Tyr, (b) L-GQD and Trp, (c) S-GQD and Phen, (d) S-GQD and Gly, (e) F-GQD and Cys, and (f) CS-GQD and Glu. Acid.

S11: Computational model for HOMO-LUMO level for GQDs with their selective amino acids

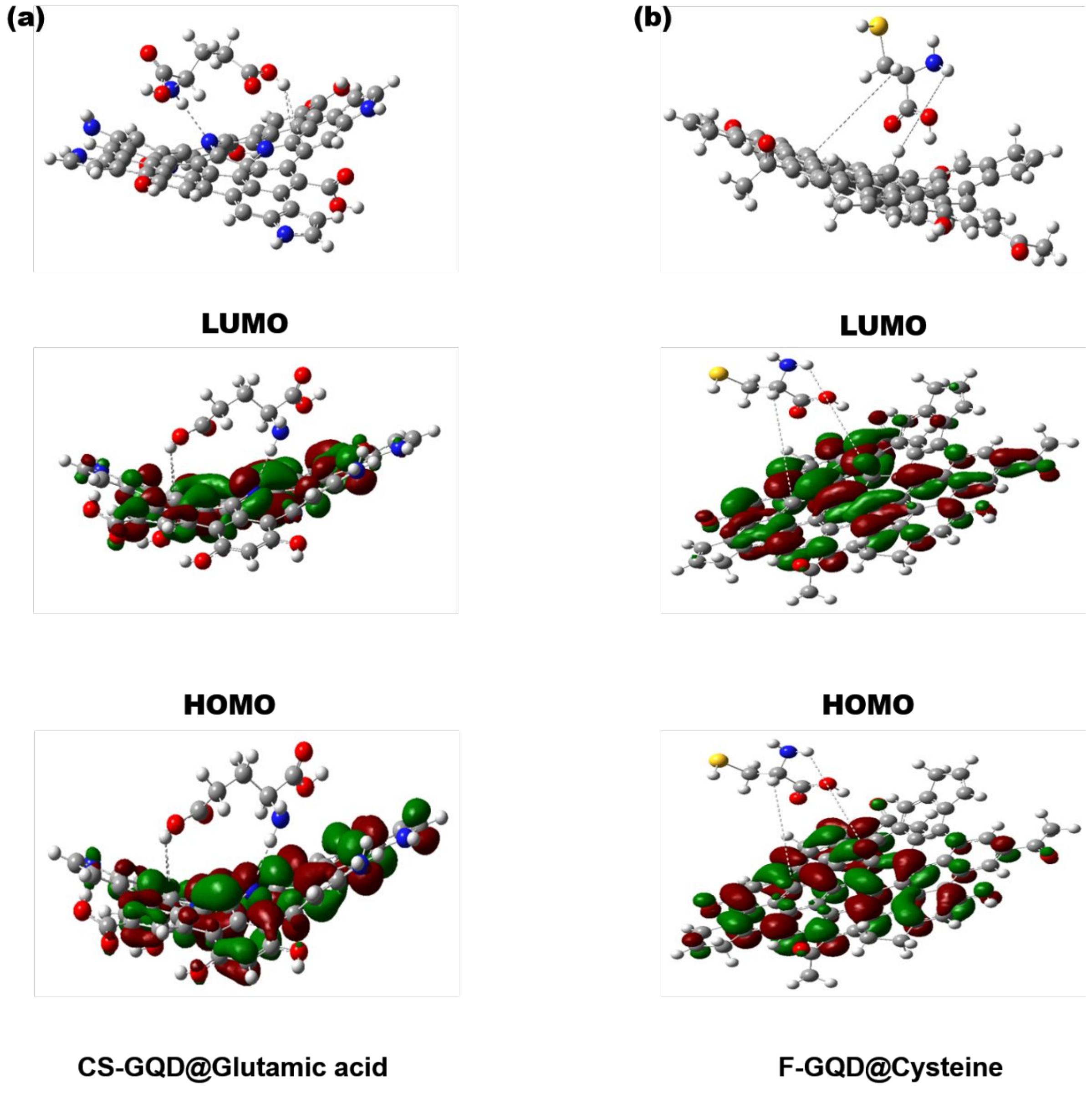

Figure S11. Computational model for HOMO-LUMO level for charge transfer between energy levels of amino acids and GQDs (a) for CS-GQD and Glutamic Acid (b) F-GQD and Cysteine.

S12: Computational model for HOMO-LUMO level for GQDs with their selective amino acids

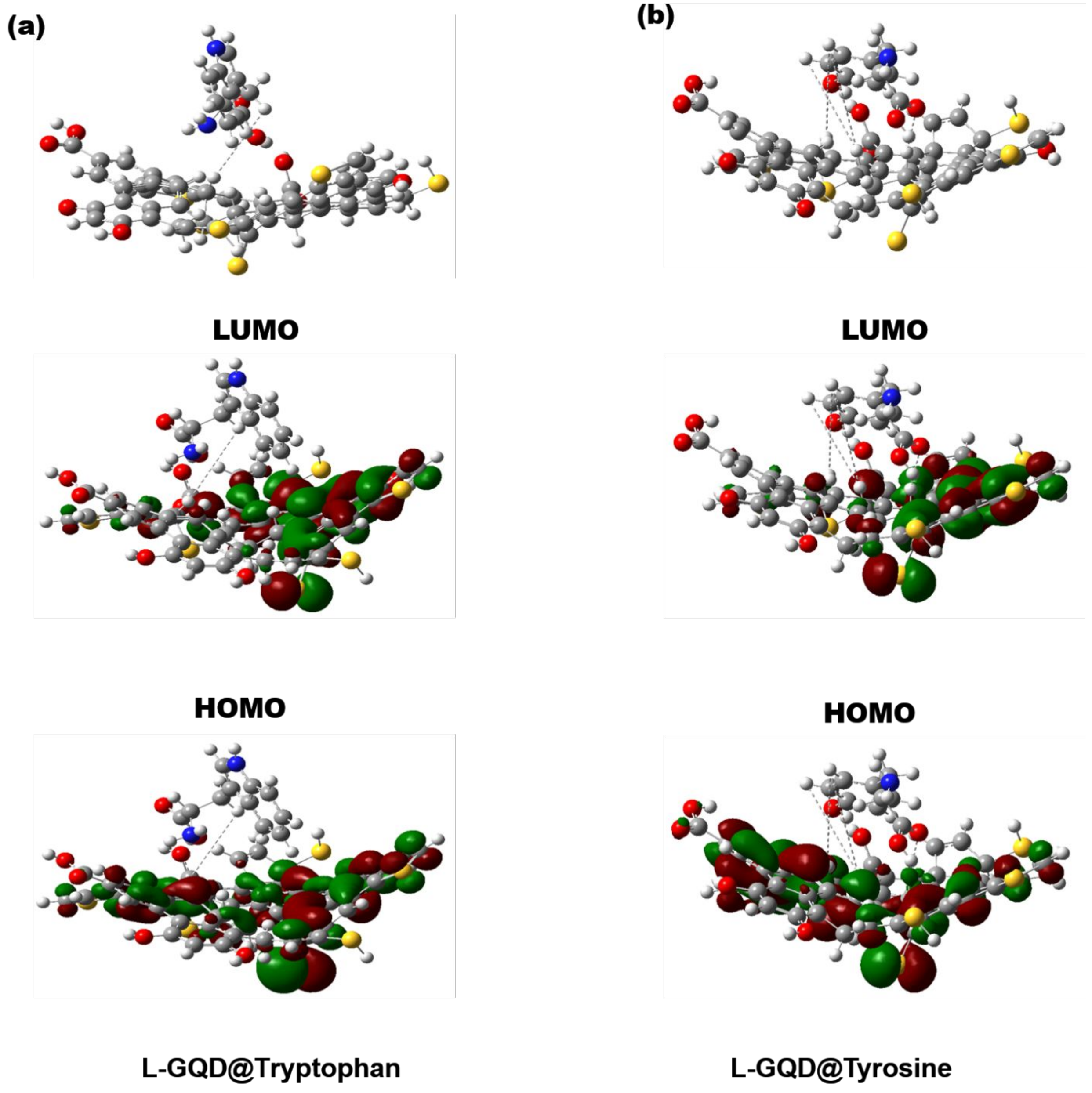

Figure S12. Computational model for HOMO-LUMO level for charge transfer between energy levels of amino acids and GQDs (a) for L-GQD and Tryptophan (b) L-GQD and Tyrosine.

S13: Computational model for HOMO-LUMO level for GQDs with their selective amino acids

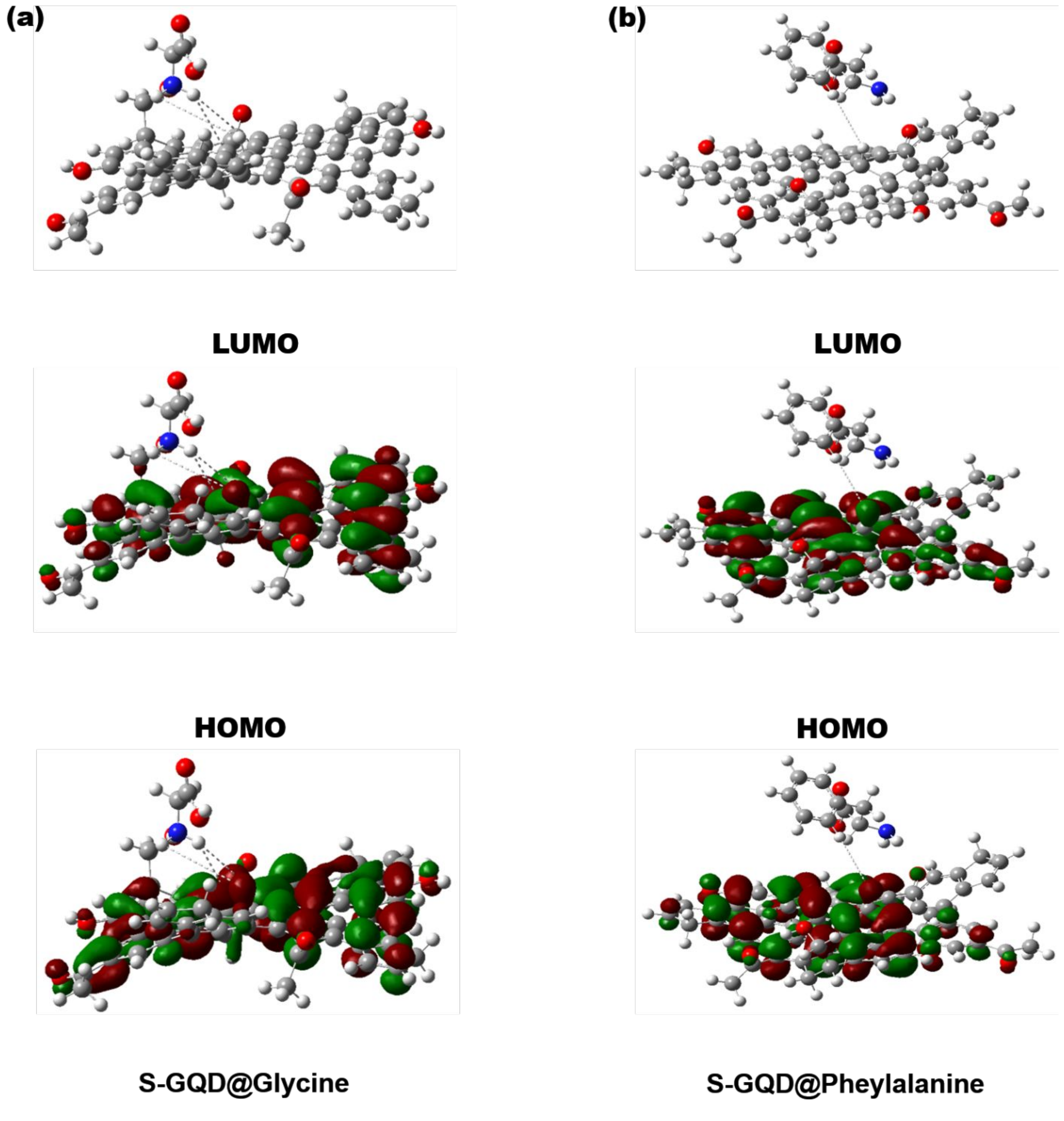

Figure S13. Computational model for HOMO-LUMO level for charge transfer between energy levels of amino acids and GQDs (a) for S-GQD and Glycine (b) S-GQD and Phenylalanine.

S14: Raman Spectra result for amino acids with GQD SERS substrates and without GQD SERS substrate.

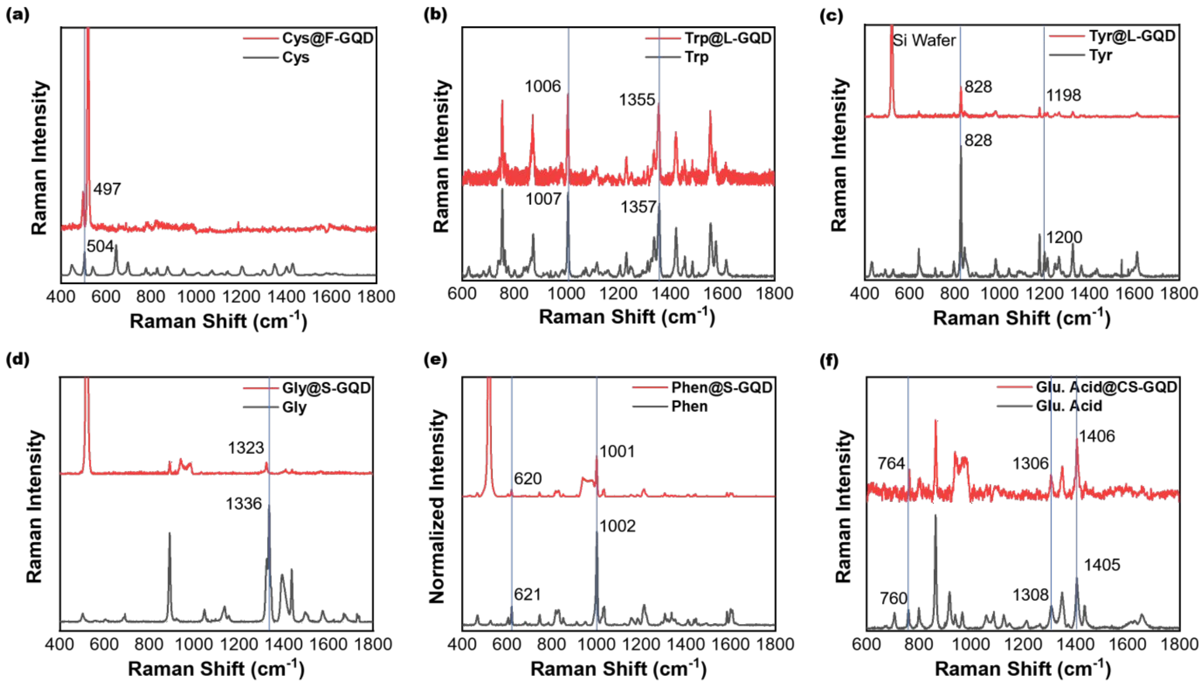

Figure S14. Raman spectroscopy study for charge transfer between GQDs and analytes. Figures showing significant peak shifts in vibrational spectra upon interaction. (a) Cys with F-GQD substrate and without F-GQD substrate, (b) Trp with L-GQD substrate and without L-GQD substrate, (c) Tyr with L-GQD substrate and without L-GQD substrate, (d) Gly with S-GQD substrate and without S-GQD substrate, (e) Phen with S-GQD substrate and without S-GQD substrate, and (f) Glu acid with CS-GQD substrate and without CS-GQD substrate.

S15: UV-Vis absorbance spectra result for amino acids with GQD SERS substrates and without GQD SERS substrate.

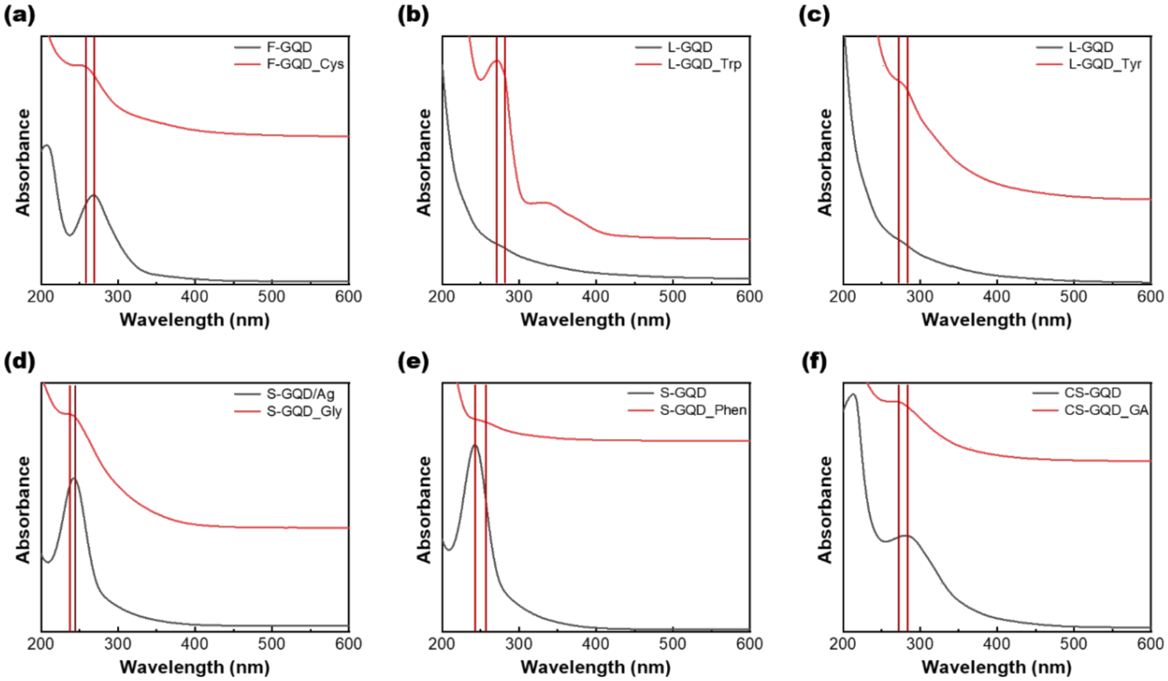

Figure S15. UV-Vis absorbance spectroscopy study for charge transfer between GQDs and analytes. Charge transfer between GQDs and their selective amino acids, revealing shifts in absorbance bands indicative of electronic interactions. UV-Vis absorbance spectra of (a) Cys with F-GQD substrate and without F-GQD substrate, (b) Trp with L-GQD substrate and without L-GQD substrate, (c) Tyr with L-GQD substrate and without L-GQD substrate, (d) Gly with S-GQD substrate and without S-GQD substrate, (e) Phen with S-GQD substrate and without S-GQD substrate, and (f) Glu acid with CS-GQD substrate and without CS-GQD substrate.

Enhancement Factor (EF) Calculation:

EF has been calculated using following formula[8]

$EF = (I_{SERS}/C_{SERS})/\ (I_{Raman}/C_{Raman})$ (S1)

Where I_{SERS} and C_{SERS} are the intensity of the significant peaks and the concentration of analyte in SERS and I_{Raman} and C_{Raman} are the intensity of the significant peaks and the concentration of analyte in normal Raman spectra.

For Example: for Trp –

I_{SERS}: 46

C_{SERS}: 10^{-4} M

I_{Raman}: 19

C_{Raman}: 10^{-3} M

$$EF = (46/10^{-4}\ M)/\ (19/10^{-3}\ M)$$

$$EF = 24.21$$

Reference

1. Rava, R. P.; Spiro, T. G., Resonance enhancement in the ultraviolet Raman spectra of aromatic amino acids. *J. Phys. Chem.* **1985,** *89*, 1856-1861.
2. Hernández, B.; Coïc, Y. M.; Pflüger, F.; Kruglik, S. G.; Ghomi, M., All characteristic Raman markers of tyrosine and tyrosinate originate from phenol ring fundamental vibrations. *Journal of Raman Spectroscopy* **2015,** *47* (2), 210-220.
3. Chuang, C. H.; Chen, Y. T., Raman scattering of L-tryptophan enhanced by surface plasmon of silver nanoparticles: vibrational assignment and structural determination. *Journal of Raman Spectroscopy* **2008,** *40* (2), 150-156.
4. Zhu, G.; Zhu, X.; Fan, Q.; Wan, X., Raman spectra of amino acids and their aqueous solutions. *Spectrochim Acta A Mol Biomol Spectrosc* **2011,** *78* (3), 1187-1195.
5. Wen, C. I.; Yu, C. N.; Thirumalaivasan, N., 532-nm-excited hyper-Raman spectroscopy of amino acids. *J Raman Spectrosc.* **2020,** *52*, 641-654.
6. Madzharova, F.; Heiner, Z.; Kneipp, J., Surface Enhanced Hyper-Raman Scattering of the Amino Acids Tryptophan, Histidine, Phenylalanine, and Tyrosine. *The Journal of Physical Chemistry C* **2017,** *121* (2), 1235-1242.
7. Jackson, A. A.; Phillips, G.; McClelland, I.; Jahoor, F., Synthesis of hepatic secretory proteins in normal adults consuming a diet marginally adequate in protein. *Am J Physiol Gastrointest Liver Physiol* **2001,** *281*, G1179-G1187.
8. Sinha, R. K., A highly sensitive surface-enhanced Raman scattering substrate prepared on a hydrophobic surface using controlled evaporation. *RSC Adv* **2021,** *12* (1), 331-337.
